# Cellular mitophagy: Mechanism, roles in diseases and small molecule pharmacological regulation

**DOI:** 10.7150/thno.79876

**Published:** 2023-01-01

**Authors:** Yingying Lu, Zhijia Li, Shuangqian Zhang, Tongtong Zhang, Yanjun Liu, Lan Zhang

**Affiliations:** 1 Sichuan Engineering Research Center for Biomimetic Synthesis of Natural Drugs, School of Life Science and Engineering, Southwest Jiaotong University, Chengdu 610031, China; 2 The Center of Gastrointestinal and Minimally Invasive Surgery, Department of General Surgery, The Third People's Hospital of Chengdu, The Affiliated Hospital of Southwest Jiaotong University, Chengdu 610031, China; 3 Medical Research Center, The Third People's Hospital of Chengdu, The Affiliated Hospital of Southwest Jiaotong University, Chengdu 610031, China

**Keywords:** Mitophagy, Mechanism, Diseases, Small molecule modulators

## Abstract

Cellular mitophagy means that cells selectively wrap and degrade damaged mitochondria through an autophagy mechanism, thus maintaining mitochondria and intracellular homeostasis. In recent years, mitophagy has received increasing attention as a research hotspot related to the pathogenesis of clinical diseases, such as neurodegenerative diseases, cardiovascular diseases, cancer, metabolic diseases, and so on. It has been found that the regulation of mitophagy may become a new direction for the treatment of some diseases. In addition, numerous small molecule modulators of mitophagy have also been reported, which provides new opportunities to comprehend the procedure and potential of therapeutic development. Taken together, in this review, we summarize current understanding of the mechanism of mitophagy, discuss the roles of mitophagy and its relationship with diseases, introduce the existing small-molecule pharmacological modulators of mitophagy and further highlight the significance of their development.

## Introduction

Mitochondria, a crucial organelle that serves as the cellular energy hub in all eukaryotic cells, is where cells carry out aerobic respiration and use oxidative phosphorylation to produce ATP 1. However, mitochondria are easy to be damaged, leading to mitochondrial dysfunction and destruction of cellular homeostasis, which is closely related to the occurrence of a variety of diseases, such as neurodegenerative diseases, cardiovascular diseases, cancer, metabolic diseases, infections and so on 2. Importantly, the damaged mitochondria need to be immediately separated and selectively removed. Therefore, mitochondrial autophagy is an important way for the body to eliminate dysfunctional mitochondria and maintain the balance of the mitochondrial environment.

**Figure 1:**
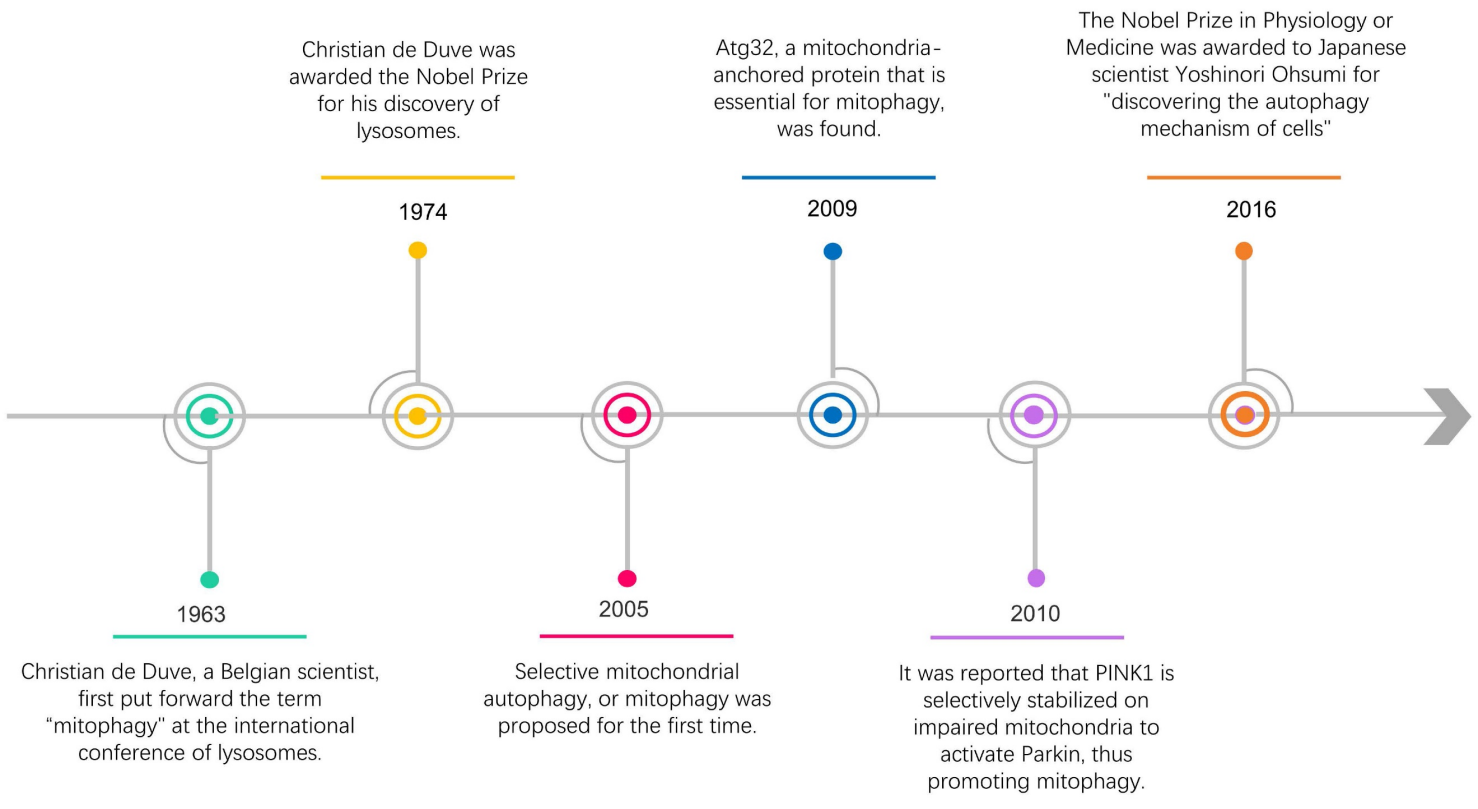
The timeline of some research progress related to mitophagy.

Almost 60 years ago, the term "autophagy" was used for the first time by Christian de Duve, who observed the degradation of mitochondria and other intra-cellular structures in the lysosomes of rat liver 3. In 2016, the Nobel Prize in Physiology or Medicine was awarded to Japanese scientist Yoshinori Ohsumi for "discovering the autophagy mechanism of cells" 4, 5 (Figure 1). Recently, along with intensive research, the scientific community has gained a deeper insight into the study of autophagy, including selective autophagy such as mitophagy. Within a few years after the word "mitophagy" was initially proposed 6, substantial developments in this field made it possible to identify crucial proteins that specifically mediate mitochondrial breakdown in yeast 7 and mammalian cells 8, 9.

**Figure 2:**
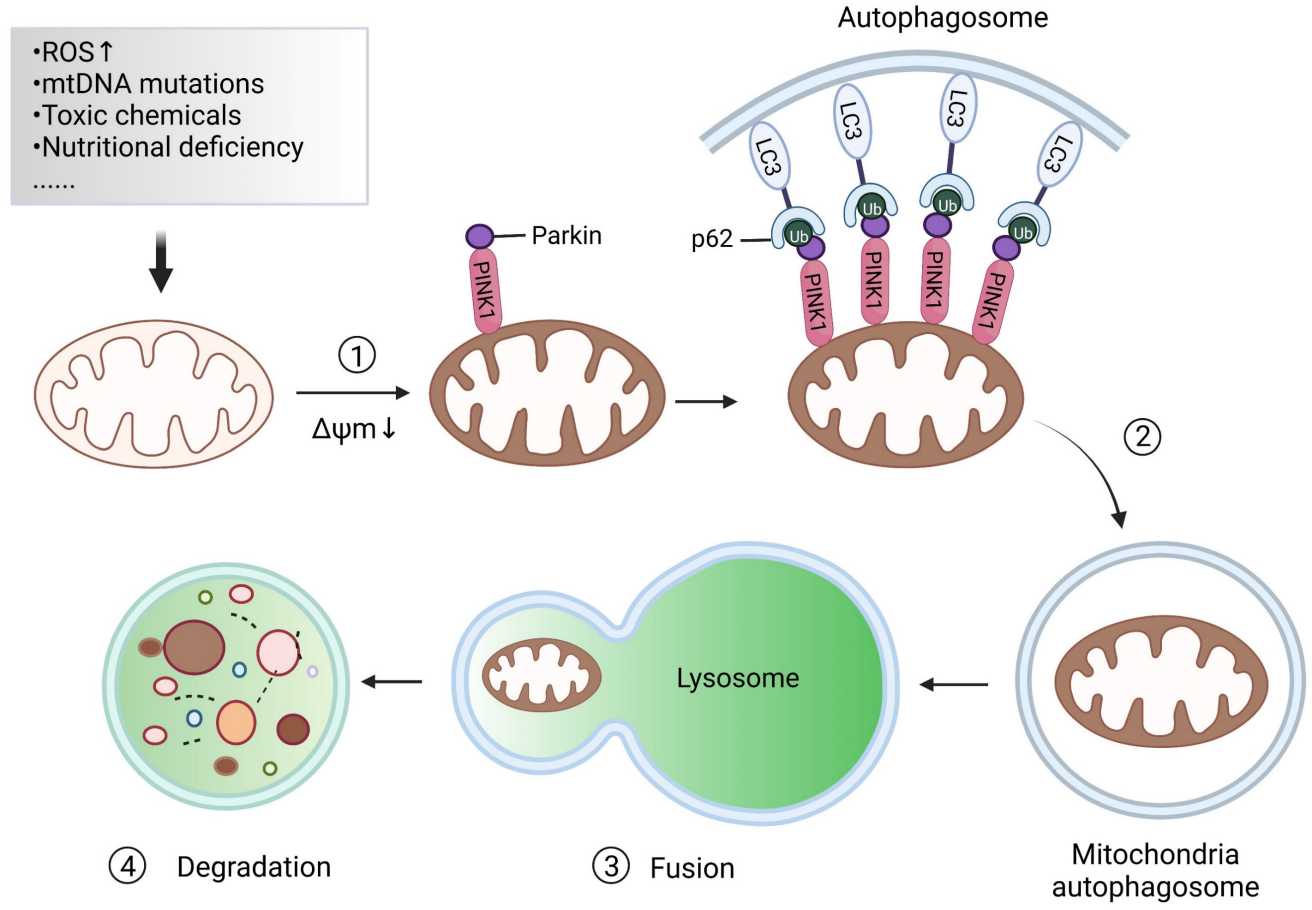
**The main processes of mitophagy.** When mitochondria are damaged after external stimulation, the damaged mitochondria will depolarize, and the outer membrane potential of the mitochondria will be lost. Subsequently, the autophagosome wraps the mitochondria to become a mitochondrial autophagosome. Lysosomes combine with mitochondrial autophagosomes, thus promoting the degradation of mitochondrial content.

Mitochondrial autophagy, also known as mitophagy, is essential for maintaining mitochondrial and cellular homeostasis 10. Under the stress of reactive oxygen species (ROS), nutrient deficiency, cell aging and other effects, the mitochondria in cells will show depolarization damage. To maintain the stability of the mitochondrial network and preserve the stability of the intracellular environment, an autophagy mechanism is used by cells to selectively wrap and degrade the damaged or dysfunctional mitochondria within the cells. This procedure is called mitophagy, which is mainly composed of four processes 11: 1) Damaged mitochondria depolarize and lose membrane potential; Under the action of ROS, nutrient deficiency, cell aging, and other external stimuli, the membrane potential of mitochondria is dissipated, which is the prerequisite for the occurrence of mitophagy. 2) Mitochondria are wrapped by autophagosomes to form mitochondrial autophagosomes; Firstly, the double-membrane phagophores that surround the mitochondria to be degraded begin to initiate and elongate. Subsequently, the phagophore is enclosed in double-membrane vesicles called autophagosomes. 3) Mitochondrial autophagosomes fuse with lysosomes; That is to say, mitochondria can be delivered to the lysosome by autophagosomes for degradation. 4) Mitochondrial contents are degraded by lysosomes. Lysosomal or vacuolar acid hydrolase flows into the autophagosome to degrade mitochondria, and the contents are recycled (Figure 2).

An in-depth analysis of the molecular mechanism of mitophagy is of great help in providing a theoretical basis for further research on the treatment of diseases related to mitophagy dysfunction. Subsequently, we discuss in detail how mitophagy plays a role in some common diseases, indicating the importance of finding new modulators targeting mitophagy. In this review, we also focus on the small molecule pharmacological modulators of mitophagy that have been reported so far, revealing the molecular mechanism of various diseases and helping to develop new drugs targeting mitophagy.

## Mechanisms of mitophagy

Since the concept of mitophagy was proposed, research on its mechanisms has received extensive attention. The mechanisms found in current studies can usually be divided into two categories: ubiquitin (Ub)-dependent pathways and Ub-independent pathways. In this section, we will put emphasis on discussing these two categories of mitophagy mechanisms.

### Ub-dependent pathways

Ubiquitin-dependent pathways, as the name implies, rely on extensive ubiquitination of mitochondrial surface proteins to promote mitophagy. Among these mechanisms, the PTEN induced putative kinase 1 (PINK1)/Parkin pathway is currently the most widely studied, which is involved in the elimination of damaged mitochondria in mammals 12. PINK1 is a highly conserved mitochondrial protein encoded by the *PARK6* gene and involved in the regulation of many cellular physiological processes, especially the regulation of mitochondrial function 13. In normal mitochondria, the expression of PINK1 is too low to be detected because it is constantly transferred to the inner membrane of the mitochondria and then removed 14. However, when the mitochondrial membrane potential (MMP, ΔΨm) is damaged, the path of PINK1 entering the mitochondrial inner membrane is blocked, resulting in PINK1 stably accumulating in the mitochondrial outer membrane. Parkin, a protein encoded by the *PARK2* gene, is an E3 ubiquitin ligase, which is responsible for connecting Ub molecules to substrate proteins, and the substrate proteins with Ub tags are recognized by proteasome and then degraded. After mitochondrial damage, the spatial conformation of Parkin changed, and cysteine, which played a catalytic role, was exposed and transformed into an activated E3 ubiquitin ligase 15. PINK1 interacts with Parkin and jointly regulate the process of mitophagy to maintain mitochondrial quality, whereas loss of either of them will cause mitochondrial damage. Previous research has shown that PINK1 and Parkin are located on the same pathway to protect mitochondria, and Parkin is located downstream of PINK1 16. Interestingly, although overexpression of Parkin can restore some of the phenotype of PINK1 deletion, PINK1 cannot compensate for the damage caused by Parkin deletion, which is probably because PINK1 is located in the upstream of Parkin 17.

**Figure 3:**
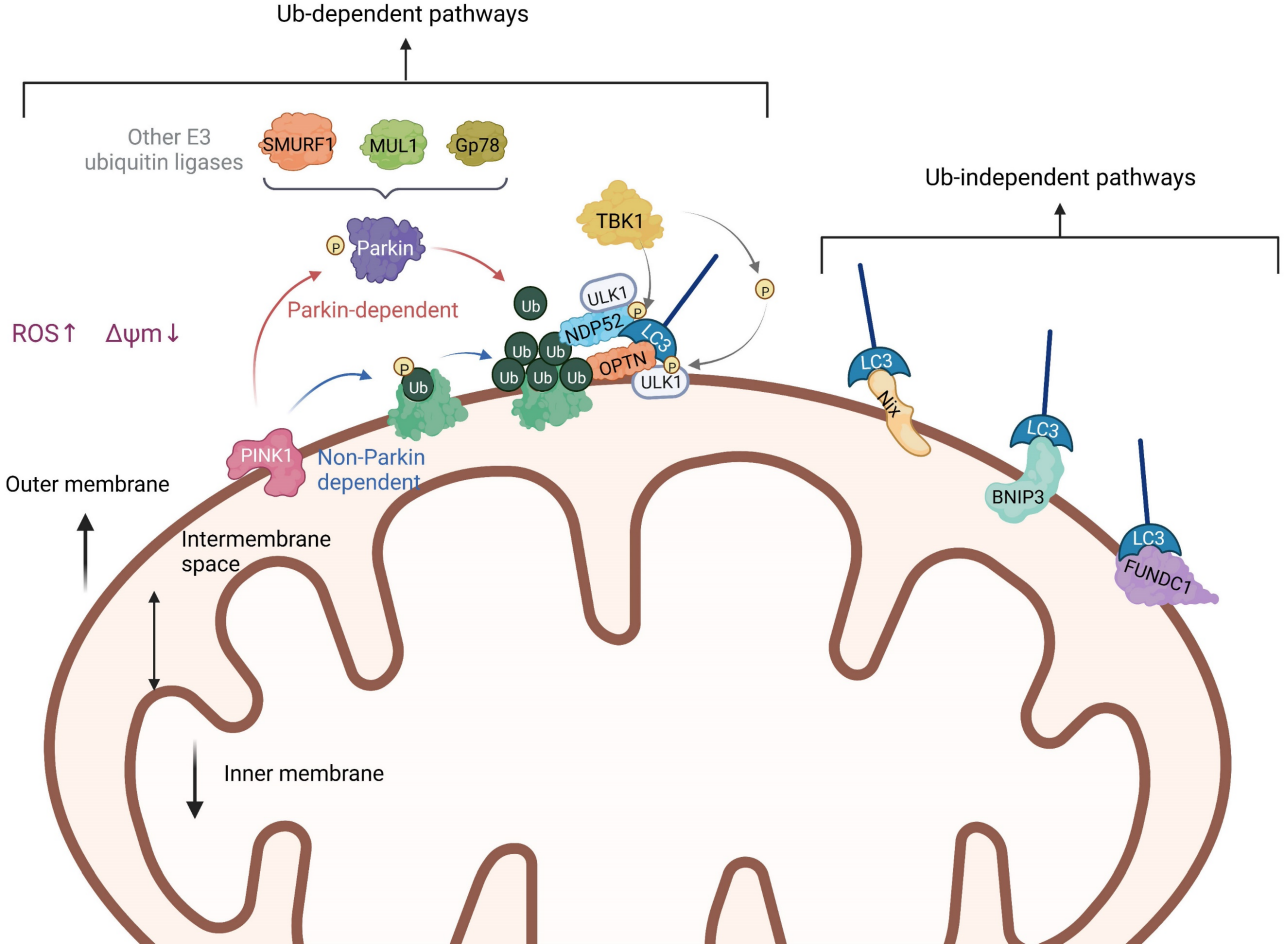
** Overview of the mitophagy mechanisms.** Mitophagy takes place through many different but interrelated mechanisms, which can usually be divided into Ub-dependent pathways and Ub-independent pathways. Among the Ub-dependent pathways, the PINK1/Parkin pathway is the most common. Besides, a series of mitochondrial autophagic receptors that can directly bind to LC3 without causing extensive ubiquitination are involved in the Ub-independent pathway.

The damaged mitochondria are commonly characterized by depolarization and a decrease in membrane potential. Recent studies have identified that PINK1 can significantly accumulate on the surface of the mitochondrial membrane with reduced potential, indicating that it may be the main detector of mitochondrial damage 18. After PINK1 accumulates and stabilizes in the outer membrane of mitochondria (OMM), it activates the E3-Ub ligase Parkin through a mechanism involving phosphorylation of Parkin and its substrate Ub at Ser65 (pSer65-Ub) 19, 20. Once located on the mitochondria, Parkin will cooperate with PINK1 to amplify the initial signal by modifying the mitochondria with the Ub chain, which is phosphorylated by PINK1 in turn (Figure 3). Sequestosome-1 (p62/ SQSTM1) is an autophagy-associated linker protein that can specifically bridge the ubiquitinated cargos into autophagosomes 21, which is involved in the process of eliminating ubiquitinated proteins and damaged mitochondria as a receptor 22. Studies have shown that p62, which preferentially locates between adjacent mitochondria in the process of PINK1/Parkin-induced mitophagy, promoted the accumulation of damaged mitochondria through its polybromo 1 (PB1) oligomeric domain 23. The knockdown of p62 had no effect on the recruitment of Parkin to the mitochondria, but affected the final elimination of damaged mitochondria. The sufficient accumulation of pSer65-Ub on the OMM can trigger the recruitment of autophagy receptors optineurin (OPTN) and nuclear dot protein 52 (NDP52), which can promote the initiation of autophagy close to mitochondria by recruiting autophagy initiation factors such as Unc-51-like kinase 1 (ULK1) 24, Double FYVE-containing protein 1 (DFCP1) and WD repeat domain, phosphoinositide interacting 1 (WIPI1) 25. Furthermore, OPTN and NDP52 can directly interact with microtubule-associated protein 1 light chain 3 (LC3) through their own LC3 interaction region (LIR) to anchor Ub-labeled mitochondria into autophagy 26, 27 (Figure 3). Importantly, Ub chains assembled by Parkin are also able to recruit autophagy receptors in complexes with a multifunctional kinase called TANK-binding kinase 1 (TBK1) 28, which also directly or indirectly mediates the phosphorylation of all known autophagy receptors. Heo et al. found that TBK1 can directly and specifically phosphorylate Ras-related protein 7A (RAB7A) to promote mitophagy through the PINK1-Parkin pathway 28. Also, a study conducted by Alban et al. discovered a self-reinforcing positive feedback mechanism that coordinates TBK1-dependent autophagy adaptor phosphorylation with the assembly of ubiquitin chains on mitochondria to facilitate efficient mitophagy 29.

In addition to the PINK1-Parkin pathway, there are ubiquitin-dependent pathways that are not Parkin-dependent. In other words, PINK1 can also directly recruit autophagy receptors OPTN and NDP52 to mitochondria through ubiquitin phosphorylation, and then promote the biogenesis of autophagy 20, 30. This indicates that OPTN and NDP52 can exist independently of Parkin. However, the presence of Parkin will amplify the signal pathway induced by PINK1 and enhance the process of mitophagy. What's more, besides Parkin, there are also other E3 ubiquitin ligases that can take part in the ubiquitination of mitochondrial proteins and the induction of mitophagy, such as smad ubiquitination regulatory factor-1 (SMURF1) 31, mitochondrial E3 ubiquitin protein ligase 1 (MUL1), Gp78 32 and so on. It has been reported that SMURF1 promotes mitophagy by recruiting defective mitochondria rather than relying on p62 33. In addition, Orvedahl et al. found that SMURF1 controls mitophagy by promoting the transfer of autophagic molecules to the generated autophagosome 31. Ryoko et al. found that MUL1 can mediate the stability of PINK1 on the mitochondrial membrane, thus inducing mitophagy, but has nothing to do with mitochondrial depolarization 34. MUL1 deficiency will increase mitofusin2 (Mfn2) activity, which will trigger the first stage of mitochondrial hyperfusion. Over-expression of Mfn2 mimics the defective phenotype of MUL1, along with the expression of protein tyrosine phosphatase interacting protein 51 (PTPIP51), an ER mitochondrial ankyrin, can inhibit Parkin-mediated mitophagy 35. Some studies have shown that Gp78 regulates mitophagy mainly by inducing mitochondrial disruption and ubiquitination, and the mitophagy induced by Gp78 is independent of Parkin 36.

### Ub-independent pathways

Unlike PINK1/Parkin-mediated ubiquitination of mitophagy, there are many proteins containing LIR regions on the OMM, which are receptors for autophagy. They can directly bind to LC3 without ubiquitination, thus initiating mitophagy. In mammals, these receptors mainly include the Nip3-like protein X (NIX)/ BCL2-interacting protein 3 like (BNIP3L) receptor, BCL2-interacting protein 3 (BNIP3) receptor, FUN14 domain containing 1 (FUNDC1) receptor, and others (Figure 3). In this part, we introduce these Ub-independent pathways in detail, namely receptor-mediated mitophagy.

NIX, also known as BNIP3L, was initially reported to be involved in mitochondrial clearance during erythrocyte maturation 9. The NIX protein can directly bind to LC3 through its BH3 domain and induce mitophagy 37. It was established that mature red blood cells from mice with NIX deficiency have mitochondria, but NIX deficiency prevents the development of red blood cells, resulting in anemia 38. Similar to NIX, BNIP3 is also located in OMM and contains a BH3 domain that can bind to LC3. Moreover, they have 56% homology because they both belong to the subfamily of the anti-apoptotic B cell lymphoma-2 (Bcl-2) family, which contains the BH3 domain 39. When BNIP3 was knocked out, the mitophagy level of neuron cells in mice was significantly reduced under hypoxia. Interestingly, the knock-out of BNIP3 can cause up-regulation of NIX expression, but this up-regulation fails to compensate for the decrease in mitophagy caused by BNIP3 knock-out 40. Furthermore, BNIP3 deficiency significantly inhibits mitophagy, thus aggravating cell apoptosis and kidney injury 41. Additionally, FUNDC1 is a mitochondrial outer membrane protein, which can induce Parkin-independent mitophagy in mammalian cells under hypoxia by interacting with LC3 42. Although FUNDC1-mediated mitophagy does not depend on Parkin, MARCH5, another mitochondrial E3 ubiquitin ligase, can regulate mitophagy under hypoxia through ubiquitin degradation of FUNDC1 43. On the other hand, in the model of cardiac ischemia/reperfusion injury, receptor-interacting serine/threonine-protein kinase 3 (RIPK3) can inhibit FUNDC1-mediated mitophagy and then promote myocardial cell apoptosis, which proves that phosphorylation is involved in regulating FUNDC1-mediated mitophagy 44. In conclusion, FUNDC1-induced mitophagy is regulated by both ubiquitin degradation and phosphorylation.

At present, the molecular regulation mechanism of mitophagy is the focus in the research field of mitophagy and cell autophagy. The effective removal of damaged or redundant mitochondria is also the key to maintain the stability of intracellular environment. Abnormal mitophagy is related to a wide array of diseases 45. Therefore, the research in this field is of great significance to reveal the pathogenesis and prevention mechanism of mitophagy-related diseases, and it is also helpful to find new drug targets as well as promote new drug development and clinical treatment.

## Roles of mitophagy in diseases

**Figure 4:**
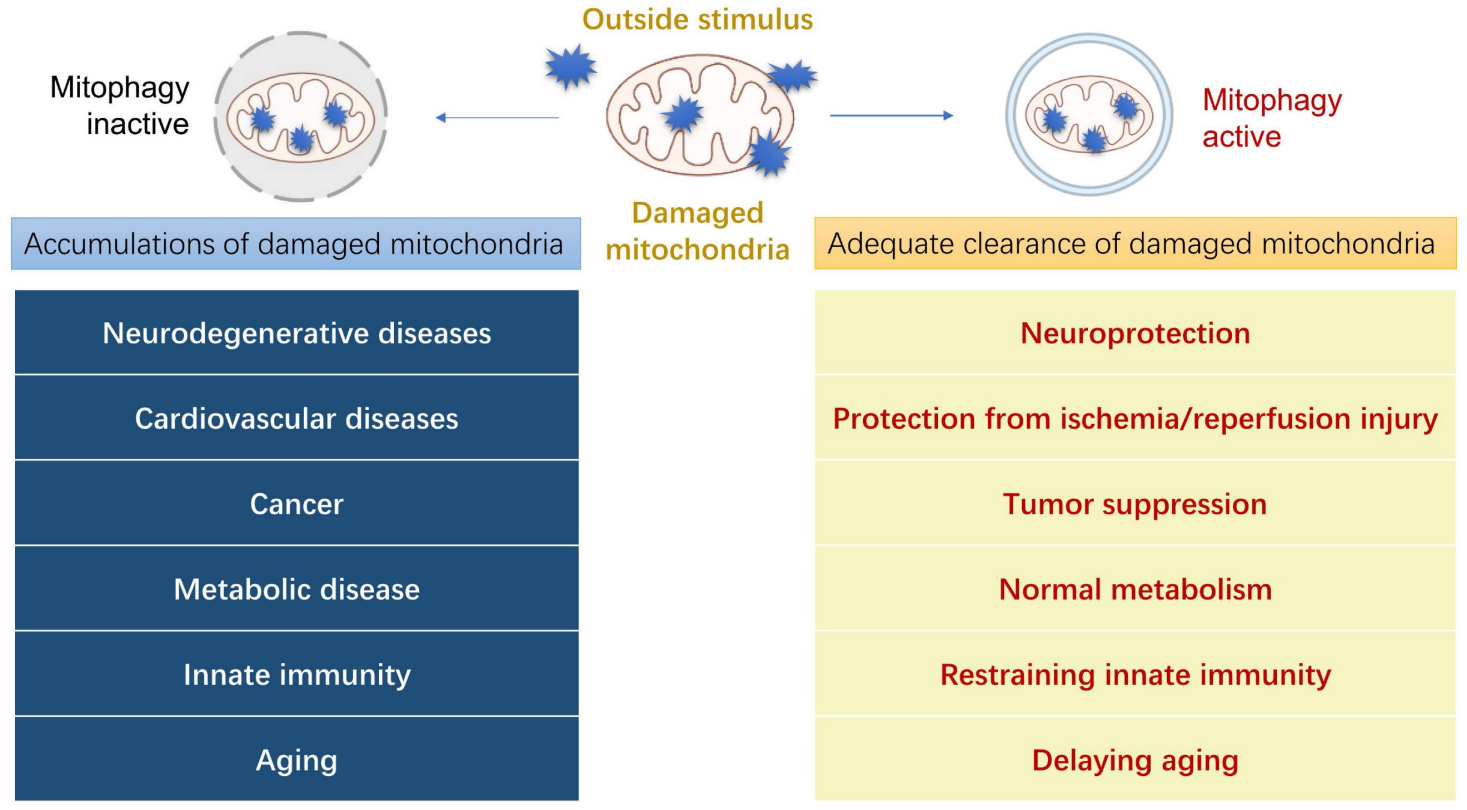
**Physiological functions of mitophagy in human diseases.** When mitophagy happens normally, it will produce a series of benefits to human body. On the contrary, the accumulation of damaged mitochondria caused by the inactivity of mitophagy will trigger some corresponding diseases.

The morphological and functional abnormalities of mitochondria are closely related to the occurrence of various diseases 46. It is highly probable that the dysfunction of mitophagy has a strong correlation with the pathogenesis of clinical diseases, leading to diseases such as neurodegenerative diseases, cardiovascular diseases, cancer, metabolic diseases, innate immunity, and other diseases (Figure 4). In this section, we shall describe how mitophagy plays a role in several prevalent illnesses and its relationship with their pathogenesis, thus providing a new potential treatment approach for these diseases by regulating mitophagy.

### Neurodegenerative diseases

Neurodegenerative diseases are closely related to age and cannot be cured at present, making it more urgent to deeply study the new pathogenesis and explore effective treatment methods. In neurons, when damaged mitochondria cannot be fully cleared by mitophagy, reactive oxygen species, nitrogen oxides, and other oxidizing substances will be produced. These oxidizing substances can bring about various neurodegenerative diseases, such as Alzheimer's disease (AD), Parkinson's disease (PD), Amyotrophic lateral sclerosis (ALS), Huntington's disease (HD), and so on 47. Although the lesion sites and causes of these diseases vary, neuronal degeneration is their common denominator. Of note, AD and PD mainly occur in the middle-aged and elderly. With the aging of the population, the incidence of AD and PD is increasing. PD is second only to AD in prevalence 48. However, ALS and HD can occur at different ages. Importantly, impaired mitophagy may give rise to the occurrence or deterioration of a series of such neurodegenerative diseases, making methods targeting mitophagy a viable therapy for these diseases.

AD, the commonest cause of dementia, is a neurodegenerative disorder that has significant implications for both individuals and society. As the largest neurodegenerative disease in the world, its prevalence continues to grow at present because the world population is still aging. Earlier studies have shown that Parkin levels in the brains of AD patients will gradually decrease with the progression of disease, which further infers that there may be a decrease of mitophagy levels in late stages of AD 49. Additionally, numerous studies have revealed that abnormal mitophagy occurs in AD patients' brains, preventing the damaged mitochondria from being cleared normally. This, in turn, accelerates pathological changes like calcium deposition and Tau protein hyperphosphorylation in AD patients' brains and worsens the onset and course of the disease 50. Notably, in both amyloid-β (Aβ) and tau C. elegans models of AD, mitophagy induced by urolithin A was demonstrated to restore memory impairment through mechanisms that dependent on PINK1, Parkinson's disease-related-1 (PDR-1) or DAF-16/FOXO-controlled germline-tumor affecting-1 (DCT-1) 50. At present, it has been proved that the progressive loss of functional nerve cells is also one of the pathological features of AD 51. For example, it has been reported that obvious neuronal apoptosis may appear in the early stage of AD 52. At the same time, deficient ATP production due to impaired mitophagy can also enhance the sensitivity of nerve cells to apoptosis-related proteins and accelerate the apoptosis of the cells. Compared with other cells in the brain, neurons are more dependent on ATP provided by oxidative phosphorylation of mitochondria 53. And dysfunction of mitochondria can directly result in the death of neurons, which affects the functions of neurons in receiving, integrating, transmitting, and conducting information 53. In brief, abnormal mitophagy induces the increase of AD-related pathological proteins such as the accumulation of Aβ and phosphorylation of Tau protein, increase the apoptosis of nerve cells, and leads to the disorder of brain energy metabolism, thus accelerating the occurrence and development of AD.

At present, there is no drug that can obviously delay or prevent AD, because it is a progressive neurodegenerative disease with multiple factors accumulated. Fortunately, in recent years, there has been evidence that mitophagy inducers can remove the abnormal mitochondria and enhance the ability of mitochondria to resist oxidative stress, namely improve mitochondrial function, thus delaying the course of AD. In the AD mouse model, supplementation of NAD^+^ precursors (such as nicotinamide) can improve mitochondrial function, reduce the deposition of Aβ and phosphorylation of Tau protein, thus improving the cognitive dysfunction of mice 53. Therefore, further exploration of the relationship between mitophagy abnormality and AD may provide a new idea for targeted induction of mitophagy regulation and improvement of neuropathological changes in AD, thereby providing a guarantee for clinical selection of treatment strategies for neurodegenerative diseases.

In addition to AD, PD is the second largest neurodegenerative disease in the world, which has no cure method. PD is characterized by the selective death of dopaminergic neurons in the substantia nigra (SN). However, because the etiology of PD is so complex, no theory has been able to fully explain the pathogenesis of PD up until this point. A series of studies have demonstrated that mitochondrial DNA (mtDNA) loss and mitochondrial damage affect individual dopaminergic neurons in PD on a regular basis, and that the autophagy function of mitochondria in the SN and amygdala of PD patients is compromised 54. Besides, late-stage PD patients can develop dementia with an accumulation of α-synuclein in Lewy bodies 55. On top of that, the mitochondria in the cells of PD animal model are enlarged and swollen, which may be caused by the wrong folding and aggregation of α-synuclein, eventually culminating in the death of dopaminergic neurons 56. All of these point to a connection between mitochondrial clearance failure and PD. Due to the identification of two proteins (PINK1 and Parkin) connected to the familial form of PD, the process of mitophagy, which is essential for maintaining neuronal homeostasis, is frequently associated with PD 57.

Studies have found that mutations in either PINK1 or Parkin can lead to the occurrence of genetic PD 20, 58. The absence of PINK1 or Parkin results in abnormal mitophagy, increased oxidative stress, and the accumulation of large amounts of toxic substances, which cause mitochondrial damage in the mesencephalic SN and eventually culminate in the death of dopaminergic neurons 8, 59. Therefore, therapeutic interventions aimed at modulating PINK1/Parkin signaling may be effective in treating PD. Notably, nitrosative stress is a significant pathogenic feature in PD 60. Parkin and PINK1 are S-nitrosylated by nitric oxide, which impairs mitophagy and causes a buildup of damaged mitochondria 61. According to previous research, Parkin KO animals had abnormal nigrostriatal pathway function, reduced synaptic excitability in spiny neurons, and an increase in extracellular dopamine concentration in the striatum 62. Parkin was phosphorylated by c-Abl kinase in mouse models of synuclein overexpression, which reduced Parkin's function and caused zinc finger protein 746 (ZNF746) to accumulate as a result. Notably, a number of studies have demonstrated that ZNF746 plays a crucial role in mediating the neurodegeneration caused by synuclein in both dopaminergic and non-dopaminergic neurons 63. Consequently, the phosphorylation of Parkin is intimately related to the development of PD. Taken together, it is certain that mitochondrial dysfunction caused by mitophagy disorder is an important factor leading to the onset of PD. Therefore, maintaining mitophagy at a normal level by using small molecule modulators will become a promising strategy for the treatment of PD, but this challenging therapeutic strategy still requires further research on the relationship between mitophagy and PD.

Although the incidence of ALS is not as high as that of AD or PD, it is a fatal and incurable disease characterized by selective degeneration of motor neurons, leading to progressive muscle weakness and death due to respiratory failure. It has been reported that inefficient turnover and aggregates of damaged mitochondria might play a role in the disease progression of ALS 64. Surprisingly, some of the ALS-related genes that have been identified so far, such as OPTN, have a functional connection to mitophagy, particularly the removal of protein aggregates or damaged mitochondria 65. Olivia et al. found that the multifunctional kinase TBK1 plays an important role in mitophagy. There are nearly one hundred different mutations in TBK1 that are related to ALS and frontotemporal dementia, which are also associated with the mitophagy receptor OPTN 66. On balance, the autophagy receptor OPTN and its kinase TBK1 are required for the selectivity of phagocytosis of damaged mitochondrial autophagosome. When OPTN is translocated to damaged mitochondria, LC3B is recruited and degraded via lysosomal fusion 67. The recruitment of p62 to damage mitochondria should be emphasized, however, it was done independently of OPTN and did not lead to the recruitment of LC3. Furthermore, the buildup of damaged or defective mitochondria is believed to be a contributing factor in the case of ALS. To note, ALS has become a public health issue because to current trends in life expectancy. Since this disease manifestly impairs mitochondrial homeostasis, autophagy and mitophagy dysregulation are among the most researched aspects of this pathology. In light of this, it may be suitable to use autophagy and/or mitophagy enhancers in the search for a cure for ALS.

Last but not least, HD is a fatal, incurable, and progressive pure genetic disease characterized clinically by seizures, chorea, cognitive decline, involuntary movement, mental retardation, dystonia, and emotional disorders 68. Studies have shown that HD is closely related to mitochondrial abnormalities. A typical example is the study on the relationship between mutant Huntington protein (mHtt) and mitochondria. Shirendeb et al. found that mHtt interacted with mitochondrial dynamin-related protein 1 (Drp1), which enhanced the activity of the GTPase Drp1, resulting in excessive fragmentation and abnormal distribution of mitochondria 69. Recent research has also demonstrated that the progression of HD can start when dead or dying mitochondria fail to be removed 70. Interestingly, grade-dependent changes in the number and size of mitochondria were observed in HD. Furthermore, Maria et al. showed that the mitochondrial structure of HD-affected neurons was damaged, smaller, and rounder, and the cristae were broken 71. In addition, research on cellular and animal models of HD shows that both nuclear and mitochondrial DNA are damaged, which further illustrates the correlation between mitophagy damage and the pathogenesis of HD 72.

Collectively, most work on mitophagy in the nervous system focuses on molecular pathogenesis and potential targets for treating nervous system diseases. However, there are still controversies around the target of mitophagy, the exact mechanism of receptor-mediated mitophagy, the differences in research and environmental conditions, whether the disadvantages of disease treatment outweigh the advantages, and how to best solve these problems. The challenge of future research is to design better models to understand the effects of mitophagy, common signal pathway, and mitochondrial defects on the pathological processes of neurodegenerative diseases. Furthermore, modulators that specifically induce or inhibit mitophagy can be used in the model to observe the changes in mitophagy and its relationship with the occurrence and development of diseases. In addition, the results of targeting mitophagy in long-term chronic diseases of the nervous system are still inconclusive, and extensive and rigorous research on short-term acute diseases of the nervous system is lacking. These problems need to be solved in future research.

### Cardiovascular diseases

In recent years, with the aging of population worldwide, the incidence and mortality rate of cardiovascular diseases have ranked first. Cardiovascular diseases refer to the diseases of heart and large vessels, which is closely related to the function of myocardial cells and mitochondrial quality control. ROS produced by cell activities and external stimulating factors can cause different degrees of damage to mitochondria, affecting the function and life span of myocardial cells. Therefore, timely and effective removal of aging and damaged mitochondria in myocardial cells plays an essential role in the survival of myocardial cells. In this part, we will discuss the relationship between mitophagy and cardiovascular diseases such as hypertension, atherosclerosis, heart failure, and myocardial ischemia/reperfusion injury respectively, providing new ideas for the clinical treatment of cardiovascular diseases.

Hypertension is a common and frequent systemic disease, which is closely related to cardiovascular diseases, cerebrovascular diseases, and kidney diseases. It is known that enhancing mitophagy helps decrease blood pressure and safeguard the cardiovascular system. Besides, the *PARK2* gene, which codes for Parkin, can be the hereditary cause of an elevation in blood pressure. According to Maurizio et al., reactivating mitophagy can lessen the risk of stroke in people with hypertension. They demonstrated that autophagy was reduced, and mitochondrial function was compromised in the brains of spontaneously hypertensive rats (SHR) on a high salt diet 73. In addition, Chen et al. found that astaxanthin can promote mitophagy and biosynthesis by increasing the expression of PINK1, Parkin, mtDNA and so on, thus reducing vascular remodeling caused by hypertension 74. However, the specific mechanism by which dysfunctional mitophagy leads to hypertension is still unclear, and further research is definitely needed.

Atherosclerosis is a common and frequently-occurring disease in the cardiovascular system. It is characterized by the formation of atherosclerotic plaques or fibrous plaques in the intima of blood vessels, and is the main cause of coronary heart disease, cerebral infarction, and myocardial infarction 75. The presence and progression of atherosclerosis are significantly influenced by oxidized low density lipoprotein (ox-LDL), while the level of mitophagy is highly correlated with ox-LDL. Evidence suggests that ox-LDL-induced apoptosis in human vascular smooth muscle cells (VSMC) was reduced by activated mitophagy. What's more, the protective impact of mitophagy on VSMC can be increased by enhancing PINK1 expression 76. In aortic endothelial cells, ox-LDL can lead to over-expression of nuclear receptor subfamily 4 group A member 1(NR4A1), which contributes to the over-activation of Parkin-mediated mitophagy 77. Therefore, atherosclerosis is aggravated by the apoptosis of endothelial cells, which is caused by an excessive reduction of mitochondria and insufficient energy supply of cells. Eukaryotic initiation factor 2α (eIF2α) aggravates hyperlipidemia-induced atherosclerotic inflammation by inhibiting the Parkin-induced mitophagy 78. To sum up, mitophagy plays a key role in the pathogenesis of atherosclerosis. It is expected to slow down or even reverse the progression of atherosclerosis by regulating this process, which may become a new target for the treatment of atherosclerosis.

Heart failure refers to a complex syndrome in which ventricular filling or ejection capacity is impaired due to cardiac dysfunction. Mitophagy is frequently blocked in the initial stages of heart failure. If the degree of mitophagy is appropriately raised, the progress of heart failure can be delayed 79. On the contrary, mitophagy is frequently overactivated in the late stages of heart failure, which leads to dysfunction of myocardial cells and aggravation of heart failure. At this time, the level of mitophagy should be properly inhibited 80. Notably, Shires et al. established different models of heart failure and confirmed that the decrease in mitophagy level can aggravate heart injury 81. Furthermore, other research has demonstrated that mice with PINK1 silenced or knocked out are more prone to reperfusion injury and pressure overload, which can result in cardiac failure 82. Actually, mitophagy disorder significantly increases ROS level and damages mtDNA, resulting in calcium overload in myocardial cells, inflammatory injury, necrosis, apoptosis, and myocardial fibrosis, all of which promote the occurrence of heart failure 83. In short, the elucidation of the role of mitophagy in the occurrence of heart failure will not only facilitate a better understanding of the pathogenesis of heart failure, but also provide more potential targets for the treatment of heart failure and have a broad application prospect.

Myocardial ischemia-reperfusion injury (MIRI) is a key problem that must be solved urgently in the treatment of ischemic myocardium. Currently, the main mechanisms of MIRI mainly include ROS, calcium overload, vascular endothelial dysfunction, mitochondrial metabolism disorder, and autophagy, which lead to apoptosis or necrosis of ischemic myocardial cells 84. The dysfunctional mitochondrial accumulation caused by insufficient mitophagy is harmful to the myocardium. And an appropriate increase in mitophagy, such as by giving therapeutic hypothermia, can alleviate MIRI 85. On the contrary, according to studies, NIX or BNIP3 is abundantly expressed in ischemic and hypoxic myocardial cells, which stimulates mitophagy. However, high NIX or BNIP3 expression can result in excessive mitochondrial clearance, which alters the energy sources of cells, and instead puts more strain on cardiac cells, producing or exacerbating myocardial ischemia-reperfusion damage 86. Therefore, the double-sided effect of mitochondria on cardiomyocytes remains to be further studied. As the relationship between the progression of MIRI in each stage and the intensity of mitophagy is still unclear, further research is definitely needed. In the future, ischemia-reperfusion injury can be alleviated and the prognosis of patients can be improved by regulating the intensity of mitophagy.

Accumulating evidence shows that mitophagy plays an irreplaceable role in maintaining the survival and normal function of myocardial cells and large vascular cells, indicating an important role in the onset, progress, and prognosis of cardiovascular diseases. Although the mechanism of mitophagy and its role in diseases have been known to some extent, the specific mechanism still needs further exploration. At the same time, as a research hotspot in recent years, the key proteins in mitophagy regulatory mechanism, such as PINK1, Parkin, BNIP3, and Drp1, are expected to become the targets of molecular therapy for cardiovascular diseases. Consequently, it is of great clinical significance to study the mechanism of mitophagy in cardiovascular diseases at molecular level.

### Cancer

Cancer, the abnormal proliferation and differentiation caused by the loss of normal regulation of local tissue cells at the gene level caused by various tumorigenic factors, is one of the main "killers" of human health at present. Studies have found that there are different levels of mitophagy in many cancers compared to normal situations 87, including rectal cancer, lung cancer, and breast cancer, reflecting the close relationship between mitophagy and cancer. Here, we discuss the roles that PINK1, Parkin, BNIP3, NIX, and FUNDC1 play in a wide array of cancers, clarifying the possible mechanisms of mitophagy in cancers.

It is conceivable that mitophagy appears to be important as a tumor-suppressive system because the buildup of defective mitochondria is engaged in carcinogenesis 88. Lauren et al. discovered that mitophagy inhibits tumor growth by removing malfunctioning mitochondria; otherwise, the abnormal mitochondria may change cells and encourage the development of tumors 89. According to Xu et al., the Warburg effect, compromised mitophagy, and easier M2 polarization of macrophages are all caused by PINK1 loss in gastric cancer 90. Furthermore, the occurrence and/or progression of cancer may be promoted by loss of heterozygosity (LOH) observed on chromosomes 6q25-q26, which can inactivate or reduce the expression of Parkin 91. Interestingly, Parkin is known to influence phosphatase and tensin homolog (PTEN)-mediated glycolytic metabolic control. Previous studies have suggested that Parkin deficiency encourages PTEN degradation, which in turn causes PI3K/AKT signaling to be activated in cancer cells. And cancer cells undergo metabolic remodeling that encourages glycolysis as a result of PI3K/AKT signaling 92. In some breast cancers, the fragile region of FRA6E containing PARK2 is often absent, which also supports the inhibitory effect of Parkin on tumor cells 93. As for the receptor of mitophagy, recent studies have shown that the increased level of BNIP3 is related to the poor survival rate of melanoma patients, while the consumption of BNIP3 in B16-F10 melanoma cells will damage the growth of tumors *in vivo* 94. High expression of NIX has been found in breast cancer, lung cancer, prostate cancer, cervical cancer, and other tumor cells. Studies have found that NIX-mediated mitophagy can promote pancreatic cancer 95. In lung cancer, high expression of NIX was also found to be associated with poor prognosis. At the same time, however, it has also been demonstrated that the damaged mitophagy caused by NIX expression deficiency in breast cancer can promote tumor metastasis 96. In conclusion, NIX may play different roles in different types of tumor cells at different stages, which may be a study hotspot in the future. According to a recent study, hepatocyte-specific FUNDC1 knockouts encourage the tumorigenesis of hepatocellular carcinoma (HCC), but FUNDC1 overexpression in hepatocytes decreases it, indicating that FUNDC1 functions to prevent HCC 97.

All in all, mitophagy, which is crucial for regulating tumor cells, is either directly or indirectly associated with the development of tumors, thus providing a potential therapy for cancer. In malignant tumors, mitophagy involves abnormal activation and proliferation of cancer cells, suggesting that it can exert both an oncogenic effect and a tumor-suppressive effect. The balance between the two effects can determine tumor progression or apoptosis 98. From this perspective, a new anticancer therapy can be formed, which not only inhibits mitophagy in cancer cells to exert the anti-cancer effect, but also enhances mitophagy in normal cells to remove damaged mitochondria and maintain the stability and normal function of mitochondrial genome to strengthen the anti-cancer effect. Therefore, further understanding of the molecular mechanism of mitophagy signal pathway is expected to provide new ideas for the formulation of clinical anti-tumor treatment strategies, which still needs further efforts by researchers.

### Metabolic diseases

Metabolic diseases are diseases caused by metabolic problems, including metabolic disorders. Currently, the most prevalent metabolic diseases include sugar metabolic diseases, lipid metabolic diseases, protein metabolic diseases, and even bone metabolic disorders. With the accelerated pace of life and changes in dietary patterns, the incidence of metabolic diseases such as diabetes and fatty liver is increasing, which has seriously endangered human health. Here, we review several common metabolic diseases that are highly related to abnormal mitophagy, providing a novel treatment strategy for these diseases.

In the past two decades, the prevalence of diabetes has been increasing to a large extent due to the aging society and the rapid increase in the obesity rate. Diabetes can cause damage to multiple organs and systems. And more than half of diabetic patients died of cardiovascular complications. Studies have shown that Parkin-mediated mitophagy can maintain pancreatic islet secretion and avoid the occurrence of type 1 diabetes (T1D) 99. The symptoms of type 2 diabetes (T2D) include hyperglycemia and insulin resistance (IR). These are invariably linked to mitochondrial damage, probably because high glucose levels boost the production of ROS from mitochondria and the oxidative stress that causes tissue damage 100. The expression of several mitophagy-related proteins, including NIX, PINK1 and Parkin, increased in early diabetic participants with mild hyperglycemia, but reduced in T2D patients 101. Interestingly, after Parkin and Atg7 knockout mice were given a high-fat diet for two months, compared to normal control mice, Atg7 and Parkin deficiency reduced mitophagy in myocardial cells, exacerbating T2D-related cardiac lesions 102. Since obesity raises the risk of T2D, diabetes and obesity are strongly related. As a result, the function that mitophagy serves in them also exhibits similarities. Obesity has also been linked to mitochondrial damage. To note, obesity-induced increases in mitochondrial content are likely due to an accumulation of damaged and fissured mitochondria that cannot be cleared by mitophagy. This is supported by the finding that obese patients have increased skeletal muscle mitochondrial content and decreased mitochondrial biogenesis 103. Furthermore, obesity is a known risk factor for liver disease. Non-alcoholic fatty liver disease (NAFLD), the largest chronic liver disease in China, is a metabolic stress liver injury that closely related to IR and genetic susceptibility. A significant contributor to the progression of NAFLD to nonalcoholic steatohepatitis is Nod-like receptor protein 3 (NLRP3). According to studies, clearing damaged mitochondria, inhibiting NLRP3 inflammatory corpuscle activation, and promoting liver lipid metabolism all depend on hepatocyte mitophagy 104. Chen et al. proved that low dose vinyl chloride can aggravate high-fat diet (HFD)-induced liver injury, which can be alleviated by PINK1/Parkin-dependent mitophagy stimulated by Alda-1 105. However, at present, the research on mitophagy and liver is still in its infancy, with few studies and inconsistent results. For example, most experimental evidence indicates that the defects of mitophagy contribute to hepatocyte steatosis and liver fibrosis 106, but some experiments also show that mitophagy is involved in the generation and differentiation of fat 107. Therefore, the relationship between the two needs to be verified by more experiments, so as to clarify how to regulate mitophagy to better treat liver diseases.

On balance, it is a very promising direction to seek new methods to treat metabolic diseases by regulating mitophagy. Also, although some studies have proved that mitophagy is closely related to metabolic diseases, the relationship between them still needs to be much further explored.

### Innate immunity

The innate immune response is a rapid response to pathogens or dangerous signals. The accurate activation of it can not only effectively eliminate pathogens, but also avoid excessive inflammation and tissue damage 108. It is well known that mitochondria are essential organelles for many biological processes, including energy production and immune response. The latest progress in immunology reveals the key role of energy metabolism in innate immune cell function 109. Therefore, maintaining the integrity and activity of mitochondrial network is a prerequisite for immunity. Here, we review the related molecular mechanism of mitophagy in immune diseases and emphasize its key role in the homeostasis of innate immune system.

Severe trauma or physical injury will lead to tissue tearing and cell damage, which will result in the release of mitochondrial danger-associated molecular pattern (DAMP) molecules (such as mtDNA) into the blood of the host 110. At this time, the immune system will start to send signals, which will cause systemic inflammatory response syndrome (SIRS), a condition characterized by fever, shortness of breath, hypotension, increased mortality, elevated heart rate, and multiple organ failure. Studies have shown that autophagy protein depletion promotes the accumulation of dysfunctional mitochondria and cytosolic translocation of mtDNA. In other words, the cytoplasmic level of mtDNA and ROS is the key factor of innate immunity activated by the inflammasome 111. In addition, the NLRP3 inflammasome is capable of detecting mitochondrial dysfunction, which may help to explain why inflammatory illnesses and mitochondrial damage are frequently linked 112. It is worth noting that mitochondria, as the central signal platform of innate immune response, have a positive feedback loop with the inflammasome. Infection destroys mitochondrial homeostasis, mediates mtDNA release, mtROS overproduction, and then triggers inflammasome stimulation, highlighting the critical role of mitophagy in the innate immune system homeostasis 113. Furthermore, as mentioned before, the regulation of mitochondrial removal is also influenced by MUL1 and SMURF1, demonstrating the immunosuppressive function of mitophagy in response to noxious stimuli.

In a word, defective removal of damaged mitochondria leads to the activation of inflammatory signaling pathways, which in turn contribute to the development of chronic systemic inflammation and inflammatory diseases. In addition, it has been reported that innate immunity can also be impaired by inducing excessive mitophagy 114. As mitophagy holds an essential role in the regulation of inflammatory responses, it is promising to develop novel methods targeting mitophagy to treat immune-related diseases.

### Other diseases

Apart from those previously discussed, mitophagy has also been demonstrated to be associated with other human diseases. Skeletal muscle atrophy is a major health problem worldwide, especially in the elderly. At present, no treatment can offset the gradual decline of skeletal muscle mass and strength with age, a process termed sarcopenia. However, more and more evidence has proved that the development of sarcopenia is caused by the accumulation of dysfunctional mitochondria, which provides a new window for exploring the treatment of this disease 115. Moreover, Harmon made the initial suggestion that ROS directly controls the aging process of cells in 1950. Linnane and Fleming both thought that mtDNA mutations and cell aging were caused by an increase in ROS production 116. However, ROS accumulation is closely related to mitophagy. For example, the knockout of PINK1 and Parkin in human bronchial epithelial cells will accelerate their aging process, accompanied by the accumulation of damaged mitochondria and an increase in ROS production 117. Besides, adipose-derived stem cells can control metabolic homeostasis and slow the aging process by encouraging mitophagy 118. Accordingly, mitophagy can remove damaged mitochondria and delay cell aging.

Although many research results to date show that mitophagy plays a key role in the development of some specific diseases, which we have summarized as much as possible here. However, there is still a requirement for more experimental research to explore the relationship between mitophagy and various diseases. We can then further develop new treatment strategies and improve the clinical therapeutic effect of these diseases by regulating mitophagy.

## Small molecules pharmacological regulation of mitophagy

Small molecules are important pharmacological tools for dissecting complex biological processes and identifying potential therapeutic interventions. As mentioned above, since mitochondrial dysfunction is associated with many types of diseases, inducing or inhibiting mitophagy is an effective therapeutic intervention strategy. Therefore, it is necessary for us to summarize the current research situation of mitophagy modulators, analyze the existing chemical tools, and discuss their advantages, limitations, as well as current applications. Here, we will review some representative small molecule pharmacological modulators of mitophagy that has been discovered so far. Small molecule mitophagy inducers can be mainly divided into targeting (Table 1) and non-targeting (Table 2), wherein the targeting can be further divided into inducers targeting mitophagy related proteins and inducers targeting non-canonical mitophagy related proteins. As to small molecule mitophagy inhibitors, the relevant study on it is relatively limited up to now, so it will not be involved too much in this section.

### Targeting small molecules mitophagy inducers

#### Small molecule inducers targeting mitophagy related proteins

**Figure 5:**
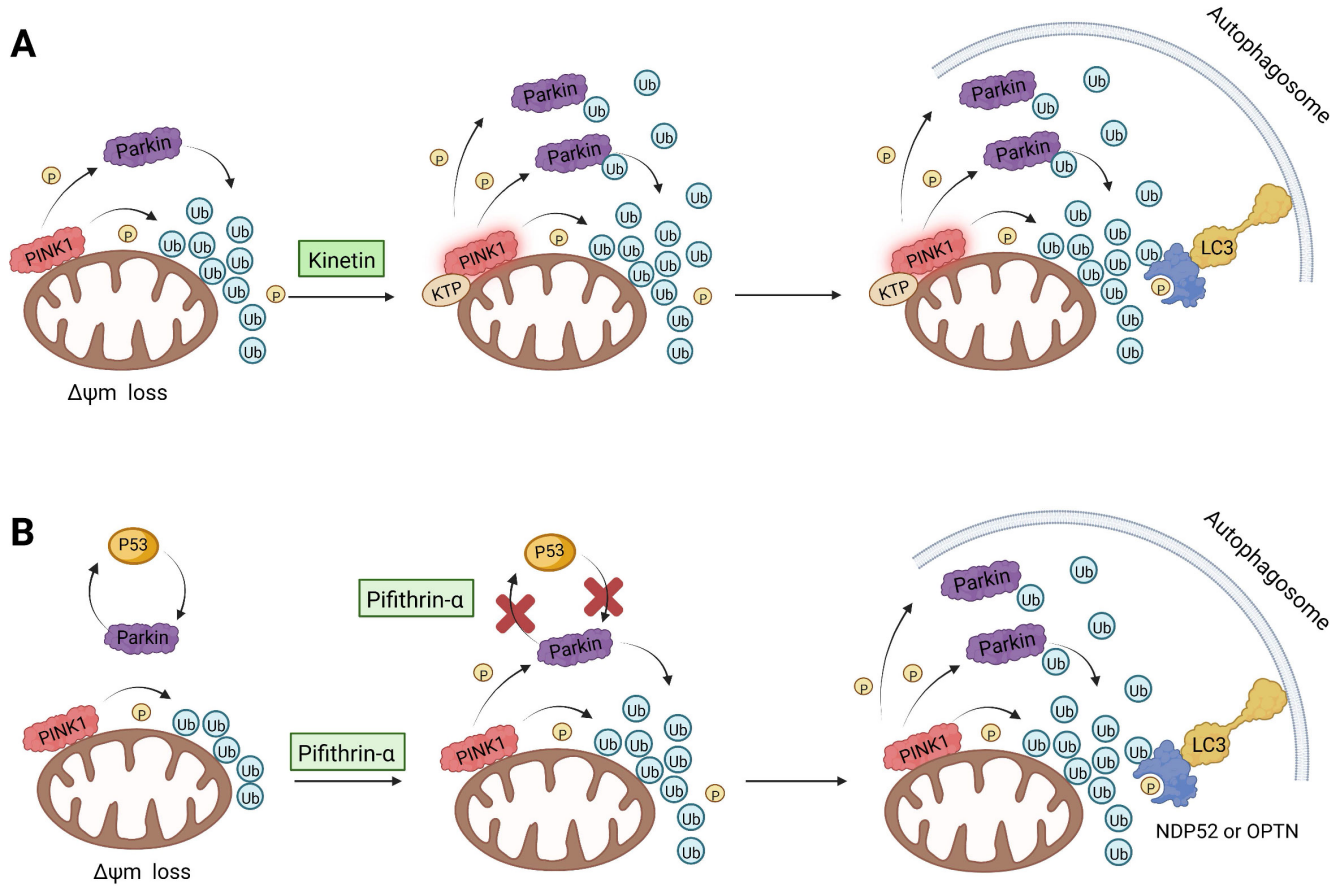
** PINK1-Parkin activity modulators.** (A) Kinetin enhanced the activity of PINK1. (B) The p53 inhibitor pifithrin-α promoted the accumulation of Parkin in the mitochondrial outer membrane.

As we mentioned earlier, the most important mechanism of mitophagy is the PINK1-Parkin pathway, so using the activity modulator of this pathway can naturally promote the occurrence of mitophagy. Previous researches have shown that kinetin triphosphate (KTP), as a new substrate, shows higher affinity for PINK1 than the natural substrate ATP 119. Interestingly, kinetin is the precursor of KTP, and its application to cells will lead to a significant increase in PINK1 activity, resulting in a higher Parkin recruitment level of depolarized mitochondria 119, 120 (Figure 5A). Therefore, kinetin and KTP are essentially PINK1 enhancers, which can promote the biogenesis of mitophagy by enhancing the activity of PINK1. In addition to PINK1 kinase, we can also regulate PINK1-Parkin pathway activity by focusing on Parkin. Previous studies have indicated that the tumor suppressor p53 may inhibit mitophagy by directly interacting with Parkin and preventing it from transferring to mitochondria 121. To note, pifithrin-α is a small molecule p53 inhibitor that can improve mitophagy by using the feedback loop between Parkin and p53 122, 123 (Figure 5B). Furthermore, it is reported that pifithrin-α can regulate Parkin expression and thus promote the occurrence of mitophagy 124. Analogously, various small molecule modulators of Parkin all have the potential to be new inducers of mitophagy.

**Figure 6:**
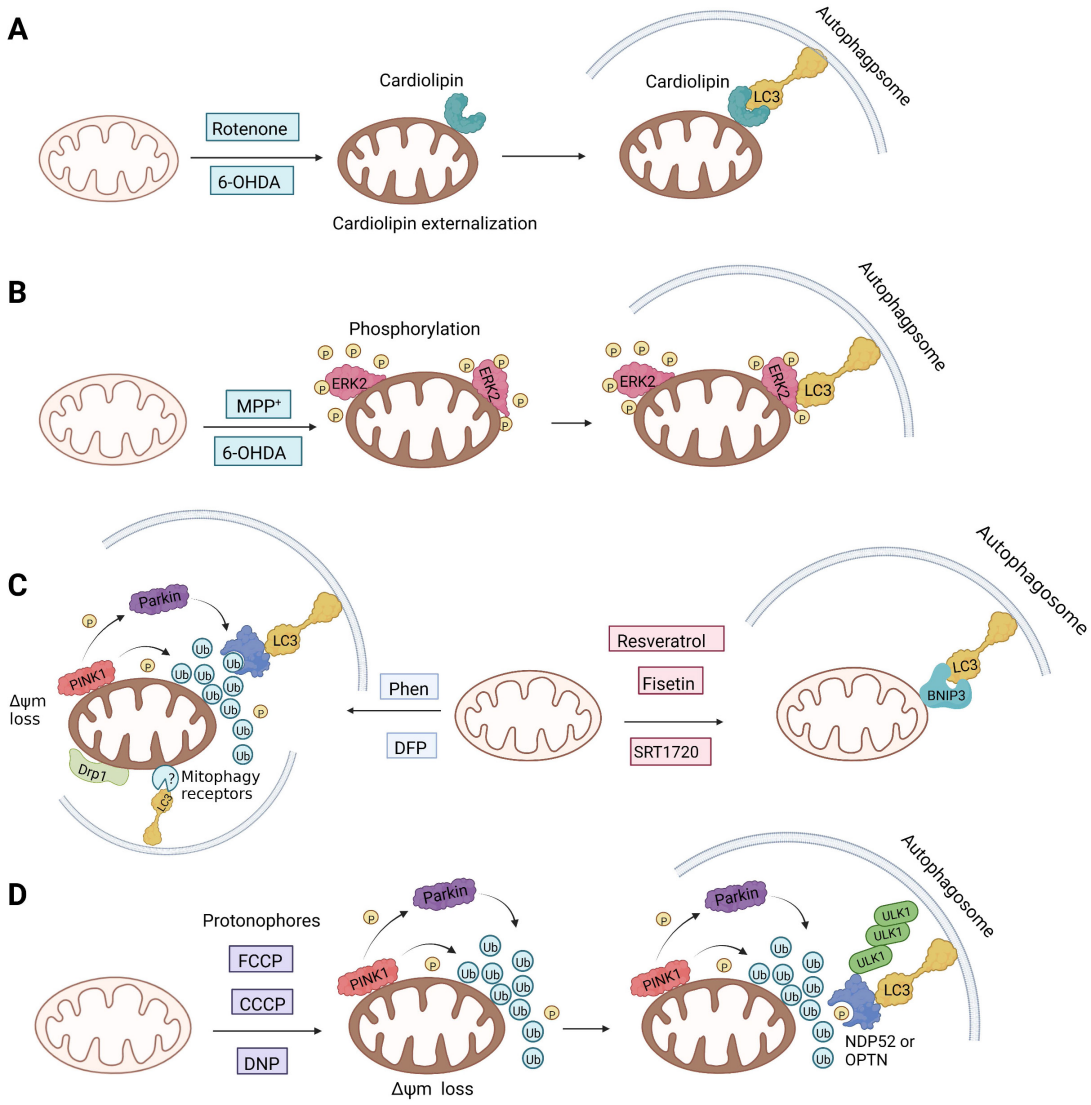
** The mechanisms of different mitophagy inducers** (A and B) Parkinson's toxins (C) Iron chelators and SIRT1 activators (D) Protonophores.

Notably, rotenone, 1-methyl-4-phenylpyridinium (MPP^+^), and 6-hydroxydopamine (6-OHDA) are all representative and significant Parkinson's toxins, which rely on ROS accumulation and mitochondrial damage to promote mitophagy 125. The distinction is that rotenone and 6-OHDA can promote the externalization of cardiolipin, which can activate the autophagy mechanism through the direct interaction with LC3 126, 127 (Figure 6A). Studies have revealed that both 6-OHDA and MPP^+^ can encourage the phosphorylation of extracellular signal regulated protein kinase 2 (ERK2) and its accumulation in mitochondria, thus promoting mitochondria to enter autophagosomes and accelerate its degradation 128, 129 (Figure 6B). In other words, rotenone induces cardiolipin-dependent mitophagy, whereas MPP^+^ induces ERK1/2-dependent mitophagy. Intriguingly, the mitophagy induced by 6-OHDA is both cardiolipin and ERK1/2 dependent 130.

1, 10-phenanthroline (Phen), an iron chelator, can act on Drp1 that is related to mitochondrial motility and induce mitochondrial fragmentation, thereby promoting mitophagy 131. Although it can mediate MMP dissipation, Park et al. found that Phen-mediated mitophagy was significantly inhibited in Drp1 knockout cells, indicating that Phen induced mitophagy in a Drp1-dependent manner 132. However, Phen treatment also enhanced autophagy, which makes its specificity for mitochondria low. Furthermore, it has mitochondrial toxicity and can cause respiratory damage. Accordingly, these shortcomings make the use of Phen as mitophagy inducers limited.

**Figure 7:**
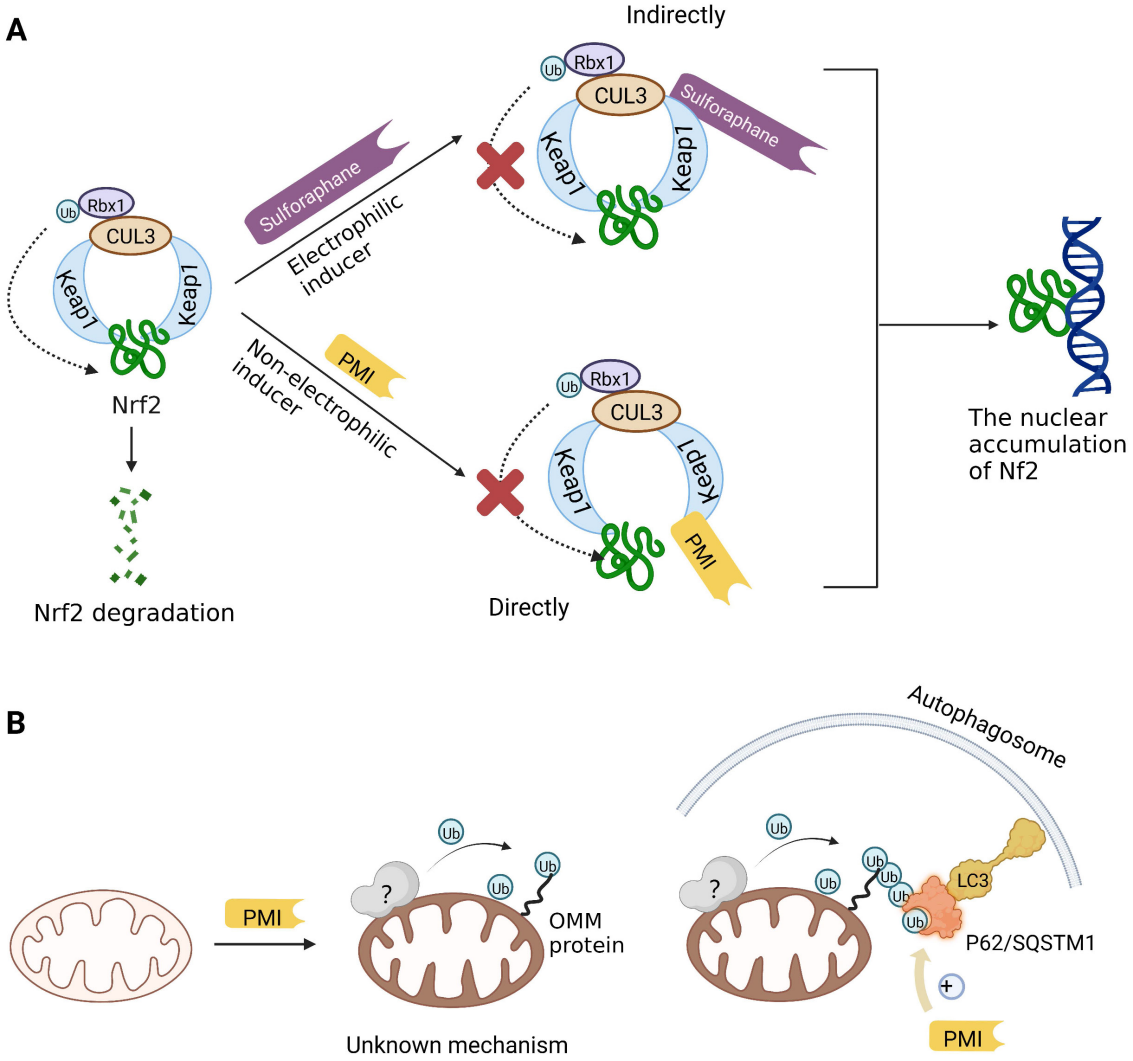
** Mitophagy inducers targeting Nrf2.** (A) Sulforaphane (indirectly) and PMI (directly) prevent Nrf2 from being degraded by Keap1, causing the nuclear accumulation of Nf2 which benefit mitochondria. (B) Nrf2 is engaged in controlling mitochondrial activity and gene transcription that activates autophagy. One such gene is p62/SQSTM1, which appears to be crucial for PMI-induced activation of mitophagy.

Previous studies have suggested that the nuclear factor erythroid 2-related factor 2 (Nrf2) is a promising target for improving mitochondrial function and health 133. Numerous advantageous effects on mitochondria are produced when Nrf2 activity is increased genetically or pharmacologically 134. A number of cell protection genes, including *SQSTM1*, which is notably connected to selective autophagy, are regulated by the transcription factor Nrf2 135. The Keap1-Nrf2 signaling pathway is currently recognized as an important defensive transduction pathway for the body to resist internal and external environmental oxidation and harmful stimulation. Nrf2 is expressed in almost all tissues, and the main factor affecting the function of Nrf2 is Keap1, which is a negative regulator of Nrf2 transcription activity and can regulate Nrf2 transcription level and post-translation level at multiple levels 136. Although there is growing knowledge of the interaction between Nrf2 and mitochondria, its function in mitophagy has just been investigated lately. Typical Nrf2 activators are electrophiles that interact with the sensor cysteines of Keap1, causing an irreversible conformational shift that prevents Nrf2 from being ubiquitinated 137. Sulforaphane, a typical example of traditional Nrf2 inducer, has been shown to be a potential therapy for cancer treatment, neurological disorders, diabetic cardiomyopathy, and other conditions 138-140. Mechanistically, sulforaphane activates mitophagy through a ROS-dependent mechanism and ERK activation 141. At the same time, it is disappointing that its non-targeted effect and low specificity limit its use. Notably, the newly found p62-mediated mitophagy inducer (PMI), a small molecule that promotes the accumulation of the Nrf2 by targeting the interaction between Nrf2 and Keap1 and inhibiting Keap1, was found to be a promising mitophagy inducer that functions without disrupting the MMP 142. PMI does not influence general autophagy but instead promotes a targeted autophagic degradation of mitochondria. In addition, compared with traditional inducers of Nrf2, PMI has a unique mode of action, which also highlights its therapeutic potential 142. Similarly, PMI and Sulforaphane prevent Nrf2 from being degraded by Keap1, causing the nuclear accumulation of Nf2 and activate transcription of genes that control autophagy and mitochondrial function (Figure 7A). However, PMI has the potential to treat diseases characterized by decreased PINK1 and Parkin activity since it may trigger mitophagy in cells lacking a fully functioning PINK1-Parkin pathway (PINK1 knockout or Parkin knockdown). And the specific mechanisms of it still need more research to figure out (Figure 7B).

Sodium selenite has been of special research interest for a long time. Since it causes human glioma cells, instead of astrocytes, to undergo a deadly form of mitophagy, which leads to irreversible cell death in glioma cells 143. The E3 ubiquitin ligase MUL1 is reportedly activated by sodium selenite in a ROS-dependent manner, which attracts ULK1 to the mitochondria and then facilitates their autophagic degradation 144. Furthermore, some studies have discovered that down-regulation of the anti-apoptotic protein myeloid cell leukemia-1 (MCL-1) can be caused by sodium selenite, thereby inducing mitochondrial-related apoptosis 145. Notably, it has been demonstrated that MCL-1 is an LC3-interacting mitophagy receptor 146. In 2014, a specific inhibitor of MCL-1 protein, UMI-77, was discovered and proved to be effective in inhibiting the growth of pancreatic cancer *in vivo* and *in vitro* 147. Later, in 2020, Cen et al. demonstrated that UMI-77 could interact with LC3A directly to promote mitophagy independent of mitochondrial damage and apoptosis. Meaningfully, they also found that using UMI-77 to target the MCL-1 protein to induce mitophagy may be a promising strategy for the treatment of AD 148.

The ubiquitin-specific peptidase 30 (USP30), a deubiquitinating enzyme found in mitochondria, is famous for its role in the regulation of mitochondrial morphology 149. By eliminating polyubiquitin chains from damaged mitochondria and hence delaying mitochondrial priming, USP30 combats Parkin-driven mitophagy 150. Importantly, USP30 knockdown or inhibition is sufficient to restore mitophagy in neuronal cells expressing a dysfunctional Parkin mutant. Consequently, USP30 inhibitors can also stimulate mitophagy by promoting normal ubiquitination. According to recent research, ST-539, a racemic phenylalanine derivative, selectively inhibited the function of the USP30 enzyme *in vitro* 151. Overexpression of USP30 prevented the decrease of the translocase of inner mitochondria membrane 23 (TIM23) and the translocase of outer mitochondria membrane 40 (TOM40) levels 149. However, ST-539 restored the degradation of TIM23 and TOM40, indicating that ST-539 can promote mitophagy by inhibiting USP30. In addition, although ST-539 promotes ubiquitination and mitophagy, PINK1/Parkin activity is imperative for the activity of ST-539 151. Disappointingly, inhibition of USP30 may lead to nonspecific events that are not directly related to mitophagy. Therefore, further research is still required to identify the rational use of USP30 inhibition as a treatment strategy for diseases.

#### Small molecule inducers targeting non-canonical mitophagy related proteins

Apart from targeting mitophagy related proteins, some small molecule that targeted non-mitophagy related proteins can also promote the biogenesis and progress of mitophagy to a high extent.

Silent information regulator T1 or Sirtuin 1 (SIRT1) is a deacetylase that regulates a variety of activities by controlling biological processes such as gene expression, DNA repair, metabolism, and mitochondrial function. In terms of mechanisms, SIRT1 may trigger mitophagy by increasing deacetylation of LC3 and then activating LC3 152 (Figure 6C). The most common activators of SIRT1 are resveratrol 153, fisetin 154, synthetic small molecule SRT1720^155^, SRT2104, SRT2379 156 and so on. They all induce mitophagy by directly activating SIRT1. However, it has recently been reported that nicotinamide (NAM) can also stimulate mitophagy by activating SIRT1 157. SIRT1 was discovered to be a NAD^+^ dependent deacetylase, whereas nicotinamide is the biosynthetic precursor of NAD^+^, implying that nicotinamide indirectly increases SIRT1 activity by increasing NAD^+^ 157. In addition to NAM, the poly (ADP ribose) polymerase-1 (PARP-1) inhibitor, such as Olaparib 158, also functions by indirectly activating SIRT1 159. Since PARP-1 is an enzyme that consumes NAD^+^, blocking it can also raise the NAD^+^ level in cells, which indirectly activates SIRT1 and then promotes the occurrence of mitophagy. In particular, although the therapeutic effect of SIRT1 activators in* in vivo* disease models has been fully demonstrated, the low specificity still restricts the use of this kind of small molecule modulator. In addition, Sirtuin-3 (SIRT3) is also an NAD^+^-dependent protein deacetylase mainly located in mitochondria 160. As the most important deacetylase in mitochondria, SIRT3 can regulate the acetylation degree of various substrates, thus affecting the malignant progress of tumors. Recently, a new small-molecule SIRT3 activator that could directly bind with SIRT3 called Compound 33c (ADTL-SA1215) was found. It has a general deacetylation activity toward the substrates of SIRT3 and depends on SIRT3 to exert its antiproliferative activity 161. Notably, studies have suggested that the Compound 33c may inhibit the metastasis and proliferation of triple negative breast cancer (TNBC) cells by promoting SIRT3-regulated mitophagy or autophagy 161. More crucially, Compound 33c may be a first-in-class selective small molecule SIRT3 activator that will be used to build new anticancer drugs.

There is also a common method of inducing mitophagy, namely respiratory complex III inhibitor and ATP synthase inhibitor. The representative drugs of this class are antimycin A and oligomycin A. By blocking respiratory complex III, antimycin A can increase the production of ROS and decrease MMP 162. Unfortunately, the decrease in MMP caused by antimycin A is relatively limited, and this decrease is quickly balanced by the reverse hydrolysis activity of ATP synthase 163. However, oligomycin A is a commonly used inhibitor of ATP synthase 164. Hence, in order to counteract this compensatory mechanism and simultaneously stimulate higher inner membrane of mitochondria (IMM) depolarization, oligomycin A and antimycin A are frequently employed in combination. Furthermore, compared to mitochondrial toxins, this method is more in line with spontaneous mitophagy and has less toxicity 20.

### Non-targeting small molecule mitophagy inducers

There are many non-targeting small molecule inducers of mitophagy, which can induce cell autophagy or other selective autophagy while inducing mitophagy. Consequently, their specificity is relatively low, limiting the possibility of clinical use.

#### Protonophores

A British academic named Peter Mitchell proposed “the chemical osmosis hypothesis” in 1961 165. He claimed that the energy released during electron transfer creates a proton gradient (H^+^ gradient) across the IMM, which powers ATP synthesis and explains how oxidation and phosphorylation are related. The energy status and operation of mitochondria can be directly measured by MMP. While mitochondrial uncoupler prevents the oxidative phosphorylation of MMP 166, it brings H^+^ from the membrane gap back to the mitochondria and releases it into the matrix in the form of protonation, thus eliminating the H^+^ concentration gradient on both sides of the mitochondrial inner membrane. Among chemical uncouplers, according to their structural properties, small molecule compounds can currently be classified as either proton carrier type or ion carrier type, with proton carrier being the most represented. In cell biology studies, these weakly acidic proton (H^+^) ionophores, also known as protonophores, are frequently utilized as an inducer of mitophagy. Typical protonophores are mainly Carbonyl Cyanide M-Chloro Phenyl Hydrazone (CCCP), Carbonyl Cyanide-P-(Trifluoromethoxy) Phenyl Hydrazone (FCCP), and 2,4-Dinitrophenol (DNP), etc. They have been frequently employed as chemical probes to stimulate mitophagy and investigate potential mitophagy mechanisms. Among them, DNP is the most concerned weakly acid uncoupler, and it is the first pharmacological application of exogenous uncoupler in human 167. In addition, CCCP, one of the earliest reported drugs to induce mitophagy, has been successfully used to reveal that PD-related protein Parkin is involved in the regulation of mitophagy 59. Notably, such protonophores induce mitophagy through the same mechanism—proton mediated dissipation of MMP (Figure 6D). Although protonophores are widely used, they still have a host of limitations in inducing mitophagy. For example, 1) the specificity is low, and they act on the entire mitochondrial population rather than a specific subgroup. Moreover, they not only have activity on mitochondrial membranes, but also on other plasma membranes, causing off-target effects. 2) They are highly toxic, and they can even lead to the complete disappearance of mitochondria in the body after long-term treatment. Namely, they have great cytotoxicity. In particular, even when the dose of FCCP is too low to cause MMP dissipation, it is already cytotoxic 168, 169. 3) This unnatural method, which does not conform to the physiological process, is harmful to mitochondria to a great extent and will lead to mitochondrial failure. Nevertheless, certain research in recent years has discovered some novel mitochondrial protonophore uncouplers with just a few drawbacks, such as BAM15, which does not depolarize the plasma membrane. BAM15 has less cytotoxicity when compared to the uncoupling agent FCCP of equal strength, and it can generate a higher maximum mitochondrial respiratory rate 170.

#### Mitochondrial toxins

Similar to protonophores, there are various toxins that can activate mitophagy following dissipation of the MMP. One of the typical mitochondrial toxins is K^+^ ionophores, such as Valinomycin and Salinomycin. Valinomycin is a respiratory chain inhibitor, and its main mechanism of activating mitophagy is to promote the inward flow of K^+^ and reduce MMP 171, 172. However, contrary to valinomycin, salinomycin can promote mitochondrial K^+^ outflow and H^+^ inflow. In other words, salinomycin causes the IMM to temporarily become hyperpolarized rather than depolarized 173. As a result, an excessive buildup of H^+^ in the mitochondrial matrix will cause the acidification that eventually results in mitochondrial failure. Another class of mitochondrial toxins that have been carefully researched includes diquat 174 and paraquat 175, 176. Diquat cause mitochondrial damage by overproducing superoxide, thus leading to mitophagy 8, 59 Similar to other toxins, paraquat-induced mitophagy is initiated by mitochondrial depolarization and operates through the PINK1-Parkin pathway.

#### Metal complexes

Metal complexes represent a promising and rapidly evolving area of pharmacotherapy. Since the serendipitous discovery of the antitumor activity of cisplatin, there has been a continuous surge in studies aimed at the development of new cytotoxic metal complexes. One of them is the metal complexes targeting mitochondria, which are able to bypass resistance mechanisms and to (re-) activate cell-death programs. Gou et al. found that some metal complexes targeting mitochondria can initiate mitophagy effectively, including organic phosphine/sulfur salts, quaternary ammonium (QA) salts, peptides, and transition-metal complexes such as guanidinium or bisguanidinium 177. Chelators are organic molecules possessing specific ligands that have high affinity, which can bind/carry metal ions and play very important roles in living systems 178. To note, one typical representative of metal complexes targeting mitochondria is iron chelators. The iron chelators can bind free iron ions, resulting in iron deficiency, and this pharmacologically induced iron deficiency can stimulate mitophagy 179. Current studies have revealed that iron loss caused by iron chelators triggers mitophagy through two or more unknown mechanisms, so further research is required to clarify these mechanisms 180. As a novel inducer of mitophagy, the iron chelator deferiprone (DFP) was found to increase mitochondrial turnover without causing MMP collapse, but through an iron-depletion-dependent mechanism 179. Hence, it does not rely on PINK1 or Parkin to play a role, but directly induces mitophagy by inducing iron deficiency 181. As for its specific mechanism, recent studies have shown that DFP may promote mitophagy through SUMO-specific protease 3 (SENP3)-mediated deSUMOylation of mitochondrial division protein Fission 1 (Fis1) 182. SUMOylation involves the attachment of a member of the small ubiquitin like modifier (SUMO) family of proteins to lysine residues in target proteins. This type of iron chelator may have therapeutic potential in inducing mitophagy, but iron chelator-induced mitophagy will result in mitochondrial toxicity, which also means respiratory damage, thus limiting the therapeutic application of such medications.

More recently, it was reported that novel glycosylation zinc (II)-cryptolepine complexes perturb mitophagy pathways and thus trigger cancer cell apoptosis and autophagy 183. Metabolism can help cancer cells escape chemotherapy, and this process mainly involves autophagy and ATP production. In view of this, Wang et al. reported a new ring metallized Ir (III) complex (Ir-Rhein) based on rhein, which can accurately target mitochondria and effectively inhibit metabolic adaptation 184. The Ir-Rhein complex can cause serious mitochondrial damage, lead to mitophagy and reduce the number of mitochondria, and then restrict the biological energy of mitochondria and glycolysis, eventually resulting in ATP starvation and death. Studies have shown that Ir-Rhein can overcome the resistance of cancer cells to cisplatin, developing a new mitochondrial-related therapy to overcome resistance to metal drugs 184.

All in all, metal complexes have attracted intense interest over recent decades as probes of mitophagy. And the further development of new metal complexes regulating mitophagy is also a very promising direction.

#### Natural products

Natural products have offered appealing alternatives for disease prevention and treatment, contributing to the development of modern drugs. Urolithin A (UA) is a kind of metabolite of ellagitannin, a polyphenol compound that is readily available and virtually ubiquitous in nature. Common foods rich in ellagic acid and ellagic tannin include pomegranates, raspberries, blueberries, walnuts, etc. 185. Additionally, UA is a new type of mitophagy enhancer, a dietary, flora-derived metabolite which can enhance muscle strength and endurance as well as mitochondrial and muscle function during aging 186. UA mainly triggers mitophagy by lowering MMP without interrupting ROS production and the mitochondrial respiratory chain. However, further study is still needed to find the specific mechanism of action. It has been shown that the enhancement of UA on mitophagy can prolong life by maintaining mitochondrial function, and it has a dose-response effect in a certain range 187.

Berberine is the main active ingredient of traditional Chinese medicine Rhizoma Coptidis and Cortex phellodendri. Its most famous function is lowering blood sugar, which has been applied to the treatment of diabetes for thousands of years 188. In addition, it also has antibacterial, gastric protection, antioxidant, anti-inflammatory, anti-hypertension and other effects 189. Liu et al. demonstrated that berberine could induce mitophagy and decrease mitochondrial ROS, thereby suppressing influenza virus-triggered NLRP3 inflammasome activation in macrophages 190. This study also confirmed that berberine can be used as a potential inducer of mitophagy.

Moustapha et al. investigated the effects of curcumin (25 μM, for 24 h) on apoptosis of human hepatoma-derived Huh-7 cells. In these cells, curcumin promote the formation of autophagic vacuoles containing degraded mitochondria and induced autophagy by increasing the expression levels of LC3-II 191. It was also found that curcumin could promote Parkin-dependent mitophagy through the AMP-activated protein kinase (AMPK)- transcription factor EB (TFEB) signal pathway, thus decrease oxidative stress-induced intestinal barrier injury and mitochondrial damage 192.

Collectively, natural products and their biological functions are currently a subject of great interest in the pharmaceutical industry, and numbers of scientific studies in this field are increasing rapidly. Dissection of the mechanisms underlying natural products that can induce mitophagy is crucial for discovering more potential therapeutic targets and promoting the development and clinical use of natural medicines.

#### Others

Based on the scaffold of a known mitophagy-promoting agent spermidine (Spd), a family of structurally related compounds were designed and tested recently. Among these compounds, Srivastava et al. found that a prototypic member, 1,8-diaminooctane (VL-004), exceeds Spd in its ability to induce mitophagy and protect against oxidative stress 193. VL-004 was shown to promote life span and health span in C. elegans and protect against oxidative injury in rodent and human cells. Moreover, Katayama et al. investigated a compound called T-271 as a promising mitophagy enhancer, which was found to act on damaged mitochondria but does not have damage on normal mitochondria 194. Additionally, Zhen et al. discovered that flubendazole, which was found to have anti-cancer effects via targeting eva-1 homolog A (EVA1A)-modulated autophagy and apoptosis before 195, could induce mitochondrial dysfunction and Drp1-mediated mitophagy by targeting EVA1A in breast cancer 196. Flubendazole increased DRP1 expression, which leads to the accumulation of PINK1 and subsequent mitochondrial translocation of Parkin, thereby promoting excessive mitophagy 196, 197.

At present, the research findings on mitophagy inducers are relatively complete and new small molecule mitophagy inducers emerge in endlessly. However, although there are many studies on inhibitors of mitophagy, the discovery of small molecule inhibitors targeting mitophagy that has high specificity is still rare, which needs further in-depth research.

### Small molecules mitophagy inhibitors

Generally, autophagy inhibitors that have been widely studied can be used to inhibit mitophagy *in vitro*. However, due to the low specificity of these inhibitors, they can also inhibit general autophagy or autophagy of other organelles besides preventing mitophagy. This also limits the use of these typical autophagy inhibitors to stimulate mitophagy *in vivo*. Therefore, in this part, we mainly introduce some small molecules that specifically inhibit mitophagy (Table 3).

Although the application of pharmacological inhibitors of mitophagy has been limited so far, new methods to specifically block mitophagy have been described. Notably, the necessary prerequisite for the initiation of mitophagy is mitochondrial division, which is mediated by the interaction between mitochondrial division protein Fis1 and Drp1 198. As Drp1 is the main protein regulating mitochondrial division in mammalian cells 199, the occurrence of mitophagy can be blocked by using compounds that inhibit the activity of Drp1. The mitochondrial division inhibitor 1 (Mdivi-1), an allosteric modulator of Drp1, was discovered by a phenotypic screening procedure in yeast cells 200. Wu et al. found that Mdivi-1 may alleviate the blood-brain barrier damage and cell death in experimental traumatic brain injury by inhibiting autophagy dysfunction and mitophagy activation 201. According to a recent study, Yang et al. described the new analogues of strigolactones (SLs) as novel autophagy/mitophagy inhibitors against colorectal cancer cells. Among these analogues, the analogue 6 exerted high specificity against colorectal cancer cells by selectively increasing the autophagic flux while blocking the autophagosome-lysosome fusion, suggesting that it is a novel autophagy/mitophagy inhibitors with high cancer cell specificity 202. Sun et al. have demonstrated that roflumilast, a phosphodiesterase 4 Inhibitor, was able to reduce the expression of mitophagy regulator proteins Drp1 and PINK1, which shows that roflumilast had the potential to serve as a new inhibitor of mitophagy 203. Most recently, Maestro et al. identified a small molecule new mitophagy inhibitor named IGS2.7 from the MBC library. Mechanistically, IGS2.7 inhibit the expression of ULK1, thus blocking autophagy and mitophagy. Intriguingly, treating different cellular and *in vivo* models of ALS with IGS2.7 restores mitophagy to normal levels, indicating a potential and novel therapeutic approach for ALS patients 204. In addition, besides small molecules, some polypeptides, such as the important Drp1 modulator cell-permeable peptide P110, can block the transport of Drp1 to mitochondria by selectively interfering with its interaction with Fis1 205. Furthermore, some peptide inhibitors that can inhibit mitophagy have been found by previous research, such as sequences containing ser13 residues, LIR mimic peptides, a cell permeability probe, and so on 206. However, although there have been many reports related to the inhibitors of mitophagy hitherto, more research is still needed to developing small molecule pharmacological inhibitors of mitophagy that are highly specific.

Apart from synthetic small molecules inhibitors of mitophagy, some natural products can also inhibit mitophagy. Quercetogetin (QUE) is a polymethoxyflavone found in citrus peels, which has been reported to have different pharmacological effects, such as anticarcinogenic, anti-viral, anti-inflammatory, antioxidant, antithrombogenic, and antiatherogenic effects 207. Kim et al. found that QUE improved mitochondrial function in human bronchial epithelial cells and protected against cigarette smoke extract (CSE)-induced apoptosis in epithelial cells by inhibiting mitophagy 208. As a result, QUE may be an option for treating lung diseases brought on by CSE. Liensinine, a major isoquinoline alkaloid, has a wide range of biological activities, including anti-arrhythmia, anti-hypertension, anti-pulmonary fibrosis, and relaxation of vascular smooth muscle, etc 209. The latest study has revealed that liensinine blocks the fusion of mitochondrial autophagosome and lysosomes by inhibiting the recruitment of RAB7A to lysosomes. Besides downregulating RAB7A, it has also been demonstrated that liensinine could inhibit Drp1-mediated mitochondrial fission 209. Thus, it is expected to develop into a novel mitophagy inhibitor and be applied to the treatment of various associated diseases 210.

In this section, we review the present small molecules inhibitors of mitophagy, including synthetic and natural compounds. Also, we summarized some mitophagy inhibitors with great potential, such as the polypeptides mentioned above, to bring inspiration for the further development of mitophagy inhibitors. Of note, more research is still needed to find highly specific autophagy inhibitors for mitochondria.

## Conclusion and Outlook

In recent years, there has been a longstanding interest in research on mitophagy and the pathogenesis of clinical diseases. It has been found that regulating mitophagy may become a new direction for the treatment of some diseases. By deeply dissecting the molecular mechanism of mitophagy, it may provide a theoretical basis for further research on the treatment of these diseases. In many diseases, the significance and central correlation of mitophagy have been fully confirmed. Mitophagy is needed to control metabolic homeostasis or remove damaged and unnecessary mitochondria, which prevents mitochondrial dysfunction and subsequent molecular events leading to disease development, such as oxidative stress. In other words, mitophagy modulators, particularly activators or inducers, may have great therapeutic potential in diseases. Accordingly, it is imperative to continue researching how diseases are impacted by mitophagy. To thoroughly explore the potential mitophagy regulatory factors, which are not only limited to proteins but also non-coding RNA, the use of some omics techniques, such as proteomics, transcriptomics, metabolomics or various modification omics, as well as single cell sequencing technology, is of particular importance. In order to lay the groundwork for the discovery of pharmacological small molecules targeting mitophagy, we need to further investigate the functions of these mitophagy-regulating factors in diseases.

Although compared to other fields, our understanding of the mitophagy pathway has advanced significantly, it is plainly weak in translating mechanistic research into therapeutically effective drugs. Nowadays, the traditional method of triggering mitophagy *in vitro* is to induce the collapse of MMP and the stability of PINK1 by chemical methods. Up to now, the vast majority of mitophagy inducers have been essentially mitochondrial toxins or mitochondrial uncouplers, which have numerous limitations, such as low specificity, high toxicity, and respiratory damage. More importantly, the clinical efficacy of mitophagy modulators has not yet been fully defined. According to the most recent research, it is noteworthy that the original protocols based on acute mitochondrial depolarization brought on by non-targeting substances (like FCCP) have been gradually refined into "milder" strategies (like antimycin A and oligomycin) intended to promote mitophagy in a way that is more in line with physiological requirements. Consequently, it is necessary to design new pharmacological methods to manipulate mitophagy and develop drugs to selectively activate mitophagy without interfering with other organelles. With the help of new detection, this process of finding novel modulators can be based on phenotypic screening, rational drug design based on specific target proteins, or accidental discovery based on natural products. In addition, artificial intelligence (AI) technology can also be used to boost drug discovery. Better utilization of modern assay technologies will contribute a lot to the future research into and validation of various medicines. For example, recently, a research team conducted virtual drug screening through AI technology and successfully found two natural small molecules, kaempferol and emodin, with obvious mitophagy induction, which indicated their potential for treatment of AD^211^. It is worth noting that in this study, researchers also compared this model with other methods to determine the accuracy of this model in identifying mitophagy inducers. The results show that the AI model used in this research is the most accurate, and the compounds found by other methods can't induce mitophagy in neurons. Moreover, the hit rate of the AI model used is significantly higher than that of high-throughput screening, and it is superior to other machine learning, quantitative structure-activity relationships (QSAR), and computer-aided methods.

Small molecules are splendid tools for studying and validating the therapeutic implications of mitophagy because they are easy to manage, act rapidly, and are mostly reversible 212. However, one of the main challenges is to find small molecules that selectively act on mitophagy. Therefore, while designing better mitophagy modulators, targeting of selective mitophagy pathways should be pursued, which may open up a wider therapeutic window. Just as how to accurately and reasonably apply autophagy modulators is still a difficult problem for these small molecule medicines to enter clinical application, the accurate application of existing mitophagy modulators will also be a big challenge in the future. Therefore, there are a series of "points for attention" that need to be considered when assessing chemical probes targeting mitophagy. It is crucial to make sure that the small molecules we choose to use conform to the recommended guidelines for developing tool compounds and chemical probes. Fortunately, the number of these chemicals is continuously increasing. Of note, in the process of small molecule drug delivery or drug release, some nanomaterials can be used to increase mitochondrial targeting and reduce toxicity, such as Titanium dioxide nanoparticles (TiO_2_-NPs) 213, superparamagnetic iron oxide nanoparticles (SPIO-NPs) 214, zinc oxide nanoparticles (ZnO-NPs) 215, and mesoporous silica nanoparticles (MSNPs) 216. Using nanomaterials to promote the transport of small molecules drug targeting mitochondria is also a potential and effective method to reduce the off-target effects of some small molecule modulators. In the future, still a lot of work remains to do to discover novel nanomaterials of all kinds targeting mitophagy. And how to exert the advantages of nanotechnology in drug delivery through reasonable design, construct ideal nanocarrier and dosage form, and develop nano-drugs with high-efficiency loading, tumor-specific enrichment and controlled release are the key problems that are waiting to be solved.

In summary, this paper reviews the mechanism of mitophagy, its roles in diseases, and its small molecule pharmacological regulation. It hopes to attract people's attention to new and specific mitophagy modulators and promote the discovery of the next generation of chemical probes and best methods for clinical use. As our understanding of mitophagy deepens, we will be able to select more challenging, but possibly more specific targets. In this way, a host of diseases related to dysfunctional mitophagy will harvest promising potential therapies.

## Figures and Tables

**Table 1:** Targeting small molecule mitophagy inducers

Classification	Names	Chemical structure	Target	Mechanisms	Applications	Advantages or limitations	Ref.
**PINK1 enhancer**	Kinetin	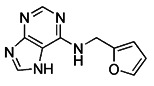	PINK1	The precursor of KTP, enhancing the kinase activity of PINK1	Preventing and treating liver fibrosis	Having no effect on the stability of PINK1 under resting conditions	119, 120
	KTP	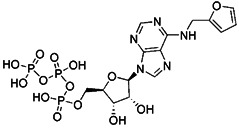	PINK1	Enhancing the kinase activity of PINK1	No application at present	Having higher affinity for PINK1 than the native substrate ATP	119
**p53 inhibitor**	Pifithrin-α	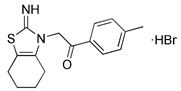	p53	Inhibiting p53 and regulating PARK2 expression	Having neuroprotection effects	Low specificity	122, 123
**Parkinson's toxin**	Rotenone	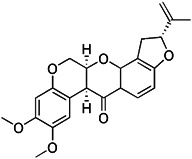	Cardiolipin	ROS accumulation and mitochondrial damage, the externalization of cardiolipin	Using as an insecticide, inducing cellular model of PD	High cytotoxicity, inducing PD	126, 127
	MPP^+^	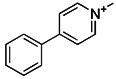	ERK2	ROS accumulation and mitochondrial damage, the phosphorylation and accumulation of ERK2	Inducing cellular model of PD	High cytotoxicity, inducing PD	129
	6-OHDA	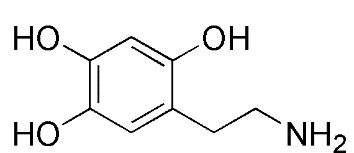	Cardiolipin, ERK2	ROS accumulation and mitochondrial damage, ERK2- and Cardiolipin- dependent	Inducing rat and cellular models of PD	High cytotoxicity, inducing PD	130
**Iron chelators**	Phen	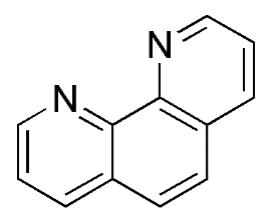	Drp1	MMP dissipation and mitochondrial fragmentation, Drp1-dependent	No application at present	Mitochondrial toxicity and respiratory damage	131
**Nrf2 inducers**	Sulforaphane	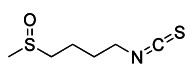	Keap1	ROS-dependent mechanism and ERK activation	Anti-cancer, protecting myocardial cells	Low specificity, a host of off-target Effects	138
	PMI	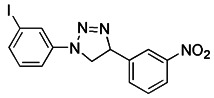	Keap1	Inhibiting Keap1	No application at present	High therapeutic potential, high specificity, further profiling would be beneficial	142
**Superoxide generator**	Sodium selenite	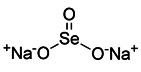	MUL1	Activating MUL1, ROS-dependent, down-regulation of MCL-1	Anti-cancer, anti-inflammation, having radioprotective effects	No toxicity, low cost, selenium supplementation	143
**MCL-1 inhibitor**	UMI-77	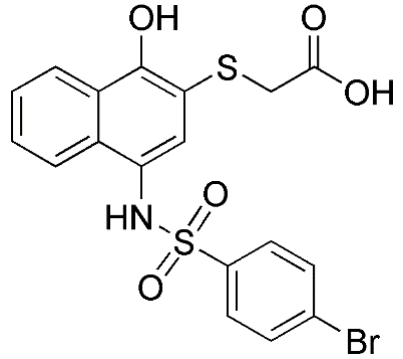	MCL1	Inhibiting MCL-1	Anti-cancer, anti-inflammation	High specificity, independent of apoptosis	147, 148
**USP30 inhibitors**	ST-539	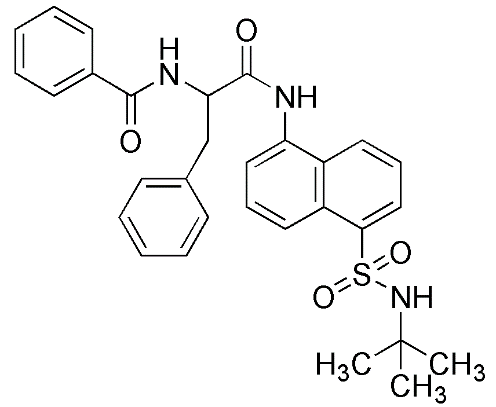	USP30	Inhibiting USP30, thus promoting ubiquitination	No application at present	Dependent of PINK1/Parkin activity, low specificity	151
**SIRT1 activators**	Resveratrol	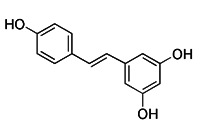	SIRT1	Activating SIRT1 directly	Anti-oxidation, anti-cancer, anti-inflammation, protecting cardiovascular system	Low specificity, also inducing general autophagy	153
	Fisetin	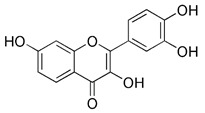	SIRT1	Activating SIRT1 directly	Anti-cancer, anti-inflammation, delaying aging	No toxicity and safe; low specificity	154
	SRT1720	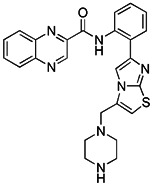	SIRT1	Activating SIRT1 directly	Anti-cancer such as bladder cancer, having neuroprotective effects, delaying aging	Having promise in clinical, the actions of it are cell specific.	155
	Nicotinamide (NAM)	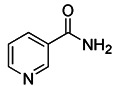	SIRT1	The precursor of NAD^+^, thus activating SIRT1 indirectly	Having neuroprotective effects, protecting skin	Without changing mitochondrial function and membrane potential	157
**SIRT3 activator**	Compound 33c	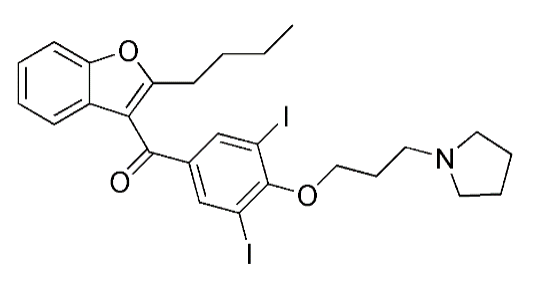	SIRT3	Activating SIRT3 directly	Anti-cancer such as TNBC	Low specificity, also inducing general autophagy	161
**PARP-1 inhibitor**	Olaparib	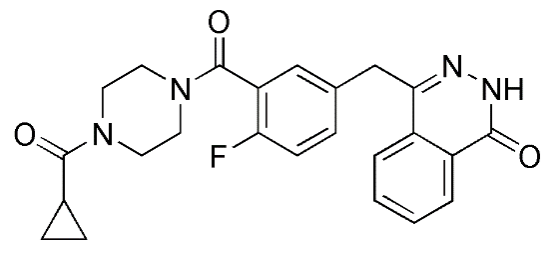	PARP-1	Inhibiting PARP-1, thus indirectly activating SIRT1	Anti-cancer such as prostate cancer, pancreatic cancer, ovarian cancer, etc.	Low specificity, inducting autophagy at the same time	158
**Respiratory complex III inhibitor**	Antimycin A	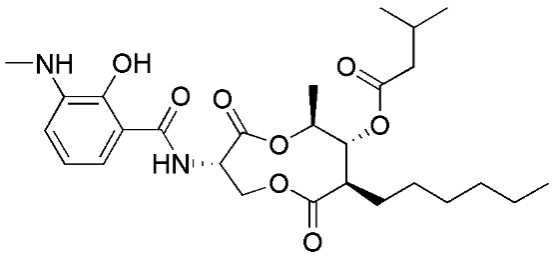	Respiratory complex III	Increased ROS levels and dissipation of MMP by inhibiting respiratory complex III	Anti-cancer such as oral cancer	Having a relatively limited effect when used alone and is frequently employed in combination with oligomycin A	162, 163
**ATP synthase inhibitor**	Oligomycin A	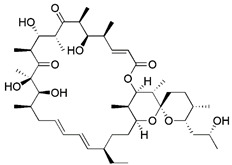	ATP synthase	Enhancing the dissipation of MMP by inhibiting ATP synthase	Anti-cancer, anti-bacteria	Having limited effect when used alone	164

**Table 2:** Non-targeting small molecule mitophagy inducers

Classification	Names	Chemical structure	Mechanisms	Applications	Advantages or limitations	Ref.
**Protonophores**	FCCP	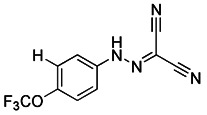	Proton mediated dissipation of MMP	Having cardioprotective effects	Low specificity, high toxicity, mitochondrial failure or even disappearance	169
	CCCP	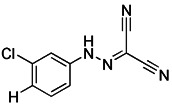	Proton mediated dissipation of MMP	No application at present	Low specificity, high toxicity, mitochondrial failure or even disappearance	59
	DNP	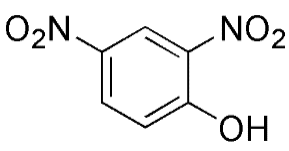	Proton mediated dissipation of MMP	Using as a weight loss agent	Low specificity, high toxicity, mitochondrial failure or even disappearance	167
**K^+^ ionophores**	Valinomycin	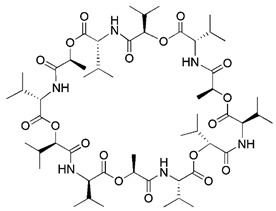	Dissipation of MMP due to K^+^ influx	Anti-virus, anti-infection	Activating the PINK1-parkin pathway without altering the pH gradient	171, 172
	Salinomycin	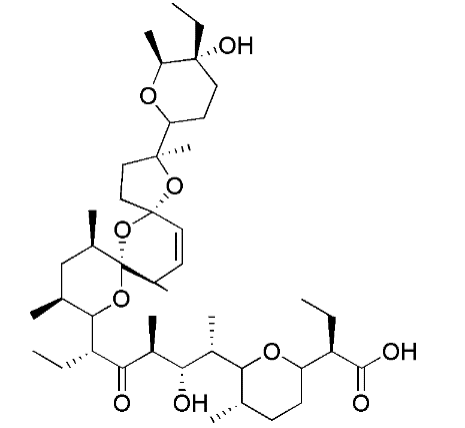	IMM is hyperpolarized due to K^+^ outflow and H^+^ influx	Anti-cancer	Mitochondrial matrix acidification and mitochondrial failure	173
**Mitochondrial toxins**	Diquat	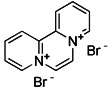	Overproducing superoxide	Using as a herbicide	High toxicity, chronic neurotoxic effects	174
	Paraquat	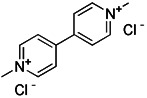	Mitochondrial depolarization	Using as a pesticide	High toxicity, an acute damage in the lung	175, 176
**Iron chelators**	DFP	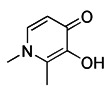	Inducing iron deficiency, iron-depletion-dependent	Anti-cancer	Mitochondrial toxicity and respiratory damage	180-182
**Metal complexes**	Glycosylation zinc (II)-cryptolepine complexes	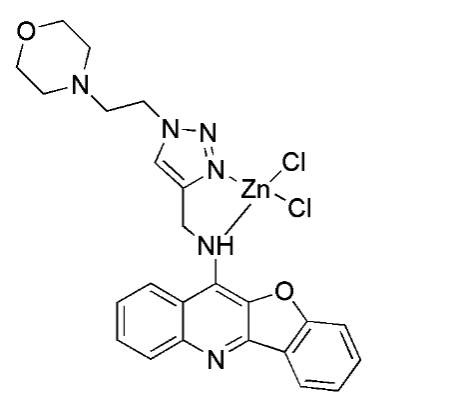	Blocking ATP production and the respiratory chain	Anti-cancer	Having potential for the development of chemotherapy drugs against cisplatin-resistant cells	183
	Ir-Rhein	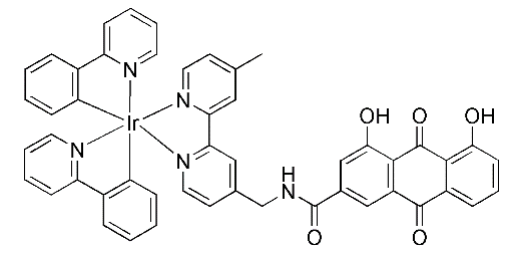	Inducing ROS elevation and mitochondrial dysfunction	Anti-cancer, overcoming metallodrug resistance	Having superior performance to overcome cisplatin resistance	184
**Natural products**	Urolithin A	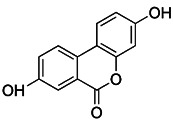	Dissipation of MMP	Anti-cancer, anti-inflammation, having neuroprotective effects, delaying aging	Easy to obtain, meriting further study	185-187
	Berberine	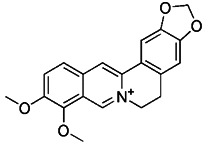	Upregulating PINK1/Parkin pathway	Anti-cancer, anti-inflammation, lowering blood sugar	Having the potential to treat various disease; side effects such as infant jaundice, and nausea and vomiting	190
	Curcumin	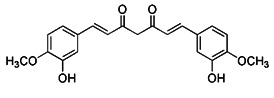	Through AMPK activation and subsequent TFEB nuclear translocation	Anti-cancer, anti-oxidative stress, anti-inflammation, having neuroprotective effects, treating osteoarthritis	Having promising roles against different diseases; excessive intake may lead to contact dermatitis	192
**Others**	VL-004	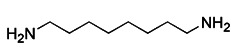	DCT-1 and PINK1- dependent mitophagy	Extending lifespan, anti-oxidative stress, having neuroprotective effects	Low toxicity, providing a structural template for the development of therapy drugs against diseases related to aging	193
	T-271	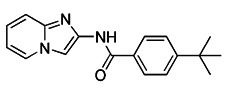	Parkin-dependent mitophagy	Treating the currently untreatable chronic progressive phases of neurodegenerative diseases	High specificity, few side effects; substantial optimization of the chemical structure is still required	194
	Flubendazole	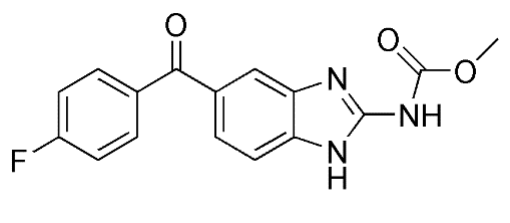	Inducing DRP1- mediated mitophagy by targeting EVA1A	Anti-cancer, treating spinal cord injury, using as an anthelmintic	Having the potential to be repurposed as a novel anti-tumor agent	196, 197

**Table 3:** Small molecule mitophagy inhibitors

Names	Chemical structure	Mechanisms	Applications	Advantages or limitations	Ref.
Mdivi-1	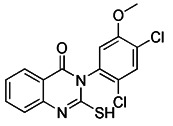	Inhibiting the activity of Drp1	Anti-inflammation, anti-oxidative stress, anti-hypertension	Low specificity, protecting cells	200
Analogue 6	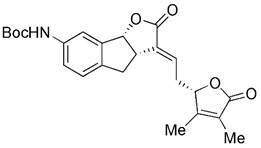	Increasing the autophagic flux while blocking the autophagosome-lysosome fusion	Anti-cancer such as breast cancer, colorectal cancer, prostate cancer, etc.	Excellent selectivity and potent cytotoxicity against cancer cells	202
Roflumilast	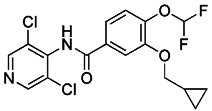	Inhibiting the expression of phosphor(p)-DRP1 and -PINK1	Treating chronic obstructive pulmonary disease (COPD), treating cognitive impairment, anti-inflammation	High security, good tolerance, few side effects	203
IGS2.7	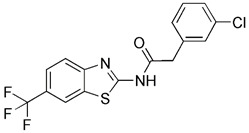	Inhibiting the expression of ULK1	Treating ALS	Having no effect on Parkin-dependent mitophagy	204
QUE	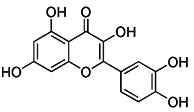	Inhibiting the expression of p-DRP1 and -PINK1	Anti-cancer, anti-inflammation, anti-virus, anti-diabetic, anti-oxidative stress	Easy to obtain, low toxicity	208
Liensinine	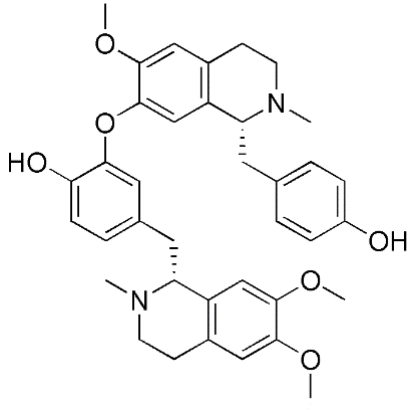	Inhibiting Drp1-mediated mitochondrial fission, blocking the fusion of mitochondrial autophagosome and lysosomes	Anti-arrhythmia, anti-hypertension, anti-cancer, anti-pulmonary fibrosis	No toxicity, having cardioprotective effects; having potential cardiac side effects	209, 210

## Abbreviations

ROS: reactive oxygen species
Ub: ubiquitin
PINK1: PTEN induced putative kinase 1
MMP/ΔΨm: mitochondrial membrane potential
OMM: outer membrane of mitochondria
p62/SQSTM1: sequestosome-1
PB1: polybromo 1
OPTN: optineurin
NDP52: nuclear dot protein 52
ULK1: Unc-51-like kinase 1
DFCP1: Double FYVE-containing protein 1
WIPI1: WD repeat domain, phosphoinositide interacting 1
LC3: microtubule-associated protein 1 light chain 3
LIR: LC3 interaction region
TBK1: TANK-binding kinase 1
RAB7A: Ras-related protein 7A
SMURF1: smad ubiquitination regulatory factor-1
MUL1: mitochondrial E3 ubiquitin protein ligase 1
Mfn2: mitofusin2
PTPIP51: protein tyrosine phosphatase interacting protein 51
NIX: Nip3-like protein X
BNIP3L: BCL2-interacting protein 3 like
BNIP3: BCL2-interacting protein 3
FUNDC1: FUN14 domain containing 1
Bcl-2: B cell lymphoma-2
RIPK3: receptor-interacting serine/threonine-protein kinase 3
AD: Alzheimer's disease
PD: Parkinson's disease
ALS: Amyotrophic lateral sclerosis
HD: Huntington's disease
Aβ: amyloid-β
PDR-1: Parkinson's disease-related-1
DCT-1: DAF-16/FOXO-controlled germline-tumor affecting-1
SN: substantia nigra
mtDNA: mitochondrial DNA
ZNF746: zinc finger protein 746
mHtt: mutant Huntington protein
Drp1: dynamin-related protein 1
SHR: spontaneously hypertensive rats
ox-LDL: oxidized low density lipoprotein
VSMC: vascular smooth muscle cells
NR4A1: nuclear receptor subfamily 4 group A member 1
eIF2α: eukaryotic initiation factor 2α
MIRI: Myocardial ischemia-reperfusion injury
LOH: loss of heterozygosity
PTEN: phosphatase and tensin homolog
HCC: Hepatocellular carcinoma
T1D: type 1 diabetes
T2D: type 2 diabetes
IR: insulin resistance
NAFLD: Non-alcoholic fatty liver disease
NLRP3: Nod-like receptor protein 3
HFD: high-fat diet
DAMP: danger-associated molecular pattern
SIRS: systemic inflammatory response syndrome
KTP: kinetin triphosphate
MPP^+^: 1-methyl-4-phenylpyridinium
6-OHDA: 6-hydroxydopamine
ERK2: extracellular signal regulated protein kinase 2
Phen: 1, 10-phenanthroline
Nrf2: Nuclear factor erythroid 2-related factor 2
PMI: p62-mediated mitophagy inducer
MCL-1: myeloid cell leukemia-1
USP30: ubiquitin-specific peptidase 30
TIM23: translocase of inner mitochondria membrane 23
TOM40: translocase of outer mitochondria membrane 40
SIRT1: Silent information regulator T1 / Sirtuin 1
NAM: nicotinamide
PARP-1: poly (ADP ribose) polymerase-1
SIRT3: Sirtuin-3
TNBC: triple negative breast cancer
IMM: inner membrane of mitochondria
CCCP: Carbonyl Cyanide M-Chloro Phenyl Hydrazone
FCCP: Carbonyl Cyanide-P-(Trifluoromethoxy) Phenyl Hydrazone
DNP: 2,4-Dinitrophenol
QA: quaternary ammonium
DFP: deferiprone
SENP3: SUMO-specific protease 3
Fis1: Fission 1
SUMO: small ubiquitin like modifier
UA: Urolithin A
AMPK: AMP-activated protein kinase
TFEB: transcription factor EB
Spd: spermidine
VL-004: 1,8-diaminooctane
EVA1A: eva-1 homolog A
Mdivi-1: mitochondrial division inhibitor 1
SLs: strigolactones
QUE: Quercetogetin
CSE: cigarette smoke extract
AI: artificial intelligence
QSAR: quantitative structure-activity relationships
TiO2-NPs: titanium dioxide nanoparticles
SPIO-NPs: superparamagnetic iron oxide nanoparticles
ZnO-NPs: zinc oxide nanoparticles
MSNPs: mesoporous silica nanoparticles
COPD: chronic obstructive pulmonary disease
